# Vaping during pregnancy: a systematic review of health outcomes

**DOI:** 10.1186/s12884-024-06633-6

**Published:** 2024-06-20

**Authors:** Michael Ussher, Joy Fleming, Leonie Brose

**Affiliations:** 1 Institute for Social Marketing and Health, University of Stirling, Stirling, FK9 4LA UK; 2 Population Health Research Institute, St George’s, University of London, London, SW17 ORE UK; 3 National Addiction Centre, Institute of Psychiatry, Psychology and Neuroscience (IoPPN), King’s College London, London, SE5 8BB UK

**Keywords:** Vaping, Electronic cigarettes, Pregnancy, Postpartum, Health consequences, Risks, Prenatal exposure

## Abstract

**Introduction:**

Smoking during pregnancy is harmful to maternal and child health. Vaping is used for smoking cessation but evidence on health effects during pregnancy is scarce. We conducted a systematic review of health outcomes of vaping during pregnancy.

**Methods:**

We searched six databases for maternal/fetal/infant outcomes and vaping, including quantitative, English language, human studies of vaping during pregnancy, to November 10th, 2023. We assessed study quality with the Mixed-Methods Appraisal Tool. We focused on comparisons of exclusive-vaping with non-use of nicotine and tobacco products and with smoking. Presentation is narrative as the studies were of insufficient quality to conduct meta-analysis.

**Results:**

We included 26 studies, with 765,527 women, with one randomised controlled trial (RCT) comparing vaping and nicotine replacement therapy for smoking cessation, 23 cohort studies and two case–control studies. While the RCT met 4/5 quality criteria, the quality of the cohort studies and case–control studies was poor; none adequately assessed exposure to smoking and vaping. For studies comparing exclusive-vaping with ‘non-use’, more reported no increased risk for vaping (three studies) than reported increased risk for maternal pregnancy/postpartum outcomes (one study) and for fetal and infant outcomes (20 studies no increased risk, four increased risk), except for birth-weight and neurological outcomes where two studies each observed increased and no increased risk. When the RCT compared non-users with those not smoking but vaping or using NRT, irrespective of randomisation, they reported no evidence of risk for vaping/NRT. For studies comparing exclusive-vaping and exclusive-smoking, most studies provided evidence for a comparable risk for different outcomes. One maternal biomarker study revealed a lower risk for vaping. For small-for-gestational-age/mean-birth-centile equal numbers of studies found lower risk for vaping than for smoking as found similar risk for the two groups (two each).

**Conclusions:**

While more studies found no evidence of increased risk of exclusive-vaping compared with non-use and evidence of comparable risk for exclusive-vaping and exclusive-smoking, the quality of the evidence limits conclusions. Without adequate assessment of exposure to vaping and smoking, findings cannot be attributed to behaviour as many who vape will have smoked and many who vape may do so at low levels.

**Study registration:**

https://osf.io/rfx4q/.

**Supplementary Information:**

The online version contains supplementary material available at 10.1186/s12884-024-06633-6.

## Introduction

Smoking during pregnancy is harmful to maternal, fetal and child health; increases the risk of adverse birth outcomes such as preterm birth, low birthweight, stillbirth, and miscarriage [1]; and is a global public health issue [1, 2]. Effective smoking cessation interventions for pregnant individuals are urgently needed. Nicotine replacement therapy (NRT) is effective for smoking cessation in non-pregnant smokers [3]; however, it is not effective in controlled trials during pregnancy [4]. This is probably due to low adherence and inadequate dosing [4]; nicotine is metabolised faster during pregnancy and standard dosing may be too low [5, 6].

Vaping (i.e., use of electronic cigarettes) provides nicotine as an aerosol [7] and is distinct from traditional NRT, allowing tailored titration of nicotine and offering flavours and enjoyment [7–9]. Consistent with the International Tobacco Control Policy Evaluation Project [10], throughout this review, we refer to these products using the term ‘vaping’. Where we use the term ‘smoking’ we are referring to combustible tobacco products. Vaping is more popular for smoking cessation than traditional NRT [11, 12]. Cessation trials, in non-pregnant populations, have shown that vaping is more effective than NRT [13]. Vaping is increasingly popular for smoking cessation and harm reduction in pregnancy [14, 15] and one randomised controlled trial (RCT) showed vaping to be more effective than NRT for cessation in pregnancy when excluding those using non-allocated products [16].

Vaping has the advantage that it does not involve tobacco combustion, which is the primary source of harm from smoking cigarettes [7, 17]. Nicotine intake from vaping has the same concerns for the fetus as nicotine from NRT. It is plausible that nicotine could detrimentally affect human fetuses. Studies with nicotine-naïve animals have reported adverse effects of nicotine on fetal lung development [18, 19]. However, caution is needed in extrapolating animal findings, using forced chronic high doses of nicotine, to intermittent self-administered nicotine intake in humans [19]. Systematic reviews and meta-analyses conclude that there is unclear evidence on whether the use of NRT during pregnancy is harmful to the fetus [20, 21]. A more recent study reported that the use of nicotine-based snuff in early pregnancy was associated with an increased risk of infant mortality; however, this study failed to consider the potential effects of smoking during the periods before and after the assessment [22].

There are also concerns about vaping ingredients other than nicotine [19]. Vaping involves heating liquid which typically contains flavourings, propylene glycol and vegetable glycerine [7], and results in the formation of carcinogens and toxicants, including nitrosamines, volatile organic compounds (VOCs), polycyclic aromatic hydrocarbons (PAHs) and heavy metals, which are generally present at much lower levels than in smoking [12, 23–25]. For the general adult population, at least in the short- to medium term, biomarker research suggests that vaping poses a small fraction of the risk of smoking [12, 25, 26]. The most recent systematic review of studies examining evidence for the health impact of vaping in pregnancy, including 13 studies published up to February 2022, reported mixed and inconclusive findings and a call for more high-quality evidence [27]. Over the last few years, there has been a proliferation of human studies on the health consequences of vaping during pregnancy and an updated review is urgently needed. We conducted a systematic review, with the principal aim of assessing evidence on health outcomes associated with exclusive-vaping in pregnancy, compared with no use of nicotine and tobacco products and compared with smoking alone.

## Methods

This review was conducted according to the Preferred Reporting Items for Systematic Reviews and Meta-Analyses (PRISMA) statement [28]. The review protocol was registered with the Open Science Framework [29].

### Eligibility criteria

We included primary, quantitative human studies, of any design, that reported on the health consequences of vaping during pregnancy and were limited to publications in peer reviewed journals in English. We excluded studies without full texts (e.g., abstract only). Although we sought all health outcomes, we anticipated including most of the important clinical outcomes for pregnant women, fetuses and infants identified in reviews of the health consequences of NTR and smoking [1, 4, 21], including miscarriage, stillbirth, preterm birth, birthweight, low birthweight, small for gestational age, admission to neonatal intensive care, caesarean section, congenital abnormalities and neonatal death. We also anticipated that biomarker studies would include most of the biomarkers of toxicants and carcinogens that have been assessed in studies of vaping among non-pregnant populations [12], including nitrosamines, VOCs, PAHs and heavy metals.

### Data sources and search strategy

We searched MEDLINE, Embase, Cumulative Index to Nursing and Allied Health Literature (CINAHL), PsycInfo, Maternity and Infant Care and the Cochrane Central Register of Controlled Trials. The searches had no start date and were conducted up to November 10th, 2023. To identify relevant systematic reviews, we also searched the Cochrane Protocols (Cochrane Library) and the International Prospective Register of Systematic Reviews (PROSPERO). We combined terms relevant to pregnancy, fetal/infant health and vaping (see Additional File 1. for search strategies). We hand‐searched references cited in the retrieved full texts. We contacted the authors of the included studies, and other colleagues working in this area, to identify studies in press but not yet published.

### Study selection and data extraction

Two authors independently screened titles and abstracts for eligibility. The full texts of relevant papers were independently assessed for inclusion by two researchers, and discrepancies were resolved by discussion between these authors and a third author if necessary. One researcher extracted information from the included studies and another researcher verified this information. Any disagreements were resolved by discussion. When the required data were not reported, they were requested from the study authors. The following data were extracted: data collection period, locality, setting, study design and aims, number of participants, participants’ characteristics, outcomes reported (including effect sizes and confidence intervals (CIs), adjusted confounders), and information on the use of nicotine and tobacco products.

### Study quality

We assessed study quality using the Mixed Methods Appraisal Tool (MMAT) [30]. We chose the MMAT because it can be applied to all types of quantitative studies. The MMAT includes five criteria. For non-randomized quantitative studies, the following criteria were used: 1. Were the participants representative of the target population? 2. Were the measurements appropriate regarding both the outcome and intervention (or exposure)? 3. Were there complete outcome data? 4. Were appropriate confounders accounted for in the design and analysis? 5. During the study period, was the intervention administered (or exposure occurred) as intended? For RCTs, the following criteria were used: 1. Was randomisation appropriately performed? 2. Were the groups comparable at baseline? 3. Were there complete outcome data? 4. Were outcome assessors blinded to the intervention provided? 5. Did the participants adhere to the assigned intervention? Two reviewers independently completed assessments and resolved discrepancies, involving a third reviewer if necessary. Calculating an overall score is discouraged; instead, as advised, we provide a table and summary of criterion ratings.

### Analysis

Meta-analysis was not considered appropriate because for none of the outcomes were there sufficient high-quality studies (i.e., at least three) [31]. Outcomes were categorised as maternal or fetal/infant and presented narratively. Maternal outcomes are reported separately for pregnancy/postpartum outcomes and for biomarkers related to maternal exposure to toxicants and carcinogens. Fetal/infant outcomes are grouped to present similar outcomes together. Where available, effect sizes are presented in the tables. For each type of outcome a summary of findings is presented in the results.

When discussing study groups, ‘exclusive vapers’ refers to those just vaping, ‘dual-users’ denotes those both smoking and vaping, ‘exclusive-smokers’ refers to those just smoking (any combustible tobacco product, although in most cases only cigarette smoking was reported), and ‘non-users’ refers to those not using nicotine or tobacco products, unless otherwise stated. When presenting the results, we focused on evidence of the risk of adverse outcomes for exclusive-vaping compared with non-use or/and compared with smoking. The risk of dual-use, compared with smoking, exclusive-smoking and non-use, is also reported but given less prominence as it is unclear how much people who vape and smoke are smoking and therefore findings are difficult to interpret.

## Results

### Study selection

**Fig. 1:**
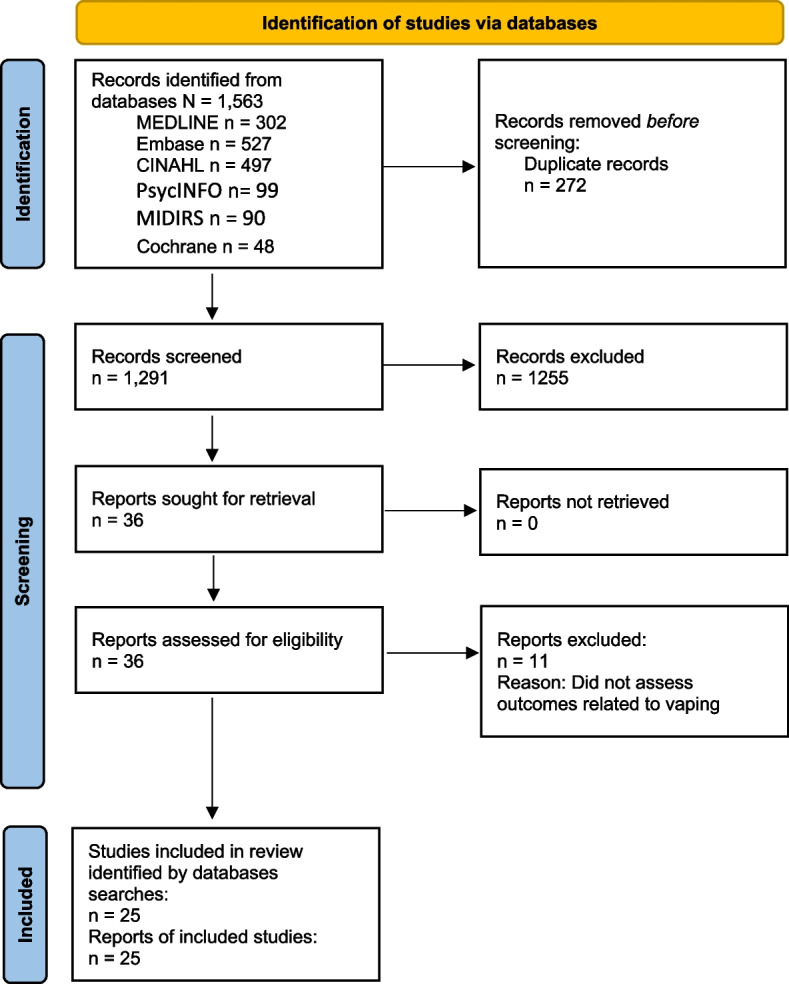
Preferred Reporting Items for Systematic Reviews and Meta‐analyses (PRISMA) flow diagram

The search identified 1,563 articles. After duplicate removal, 1,291 records were screened for eligibility. Thirty-six full texts were reviewed and 25 studies were included (see Fig. 1. PRISMA flow diagram) [16, 32–55]. In addition, one study was identified from hand‐searching references cited in retrieved full texts [56], and one ‘in press’ article was identified [57] as a secondary analysis of data from one of the studies identified in the search [16]. Therefore, a total of 26 studies, published between 2019 and 2023, were included.

### Description of included studies

Table 1. presents the study aims, design, sample size, maternal characteristics, and outcomes of the included studies, including 765,527 women, with two further studies focusing on 67 stillbirths [44] and 83 infants [40]. Table 2. presents the vaping and smoking groups and their associated characteristics.

**Table 1:** Study aim, design, maternal characteristics, and outcomes

Author, date, setting, country, timescale	Study aim	Design and sample size	Maternal age (years)	Ethnicity/race	Socioeconomic status/education/income	Time of assessment in pregnancy/postpartum	Covariates assessed	Outcomes
Ammar et al 2023 [32] National survey using Pregnancy Risk Assessment Monitoring (PRAMS) data, USA 2016–2020	To investigate the risk of vaping during pregnancy on adverse birth outcomes of PTB, LBW, and SGA, and to assess whether quitting vaping in pregnancy reduces these health risks	Retrospective cohort study, secondary analysis. Restricted to those with data on smoking and vaping in last 3 months of pregnancy *N* = 190,707 women	At delivery (weighted): ≤ 24: 23% 25–29: 29% 30–34: 30% ≥ 35: 19%	Weighted: Non-Hispanic White: 57% Non-Hispanic Black: 15% Hispanic: 19% Other: 9%	Weighted: College graduate or more: 36% Annual household income ($): ≤ 20,000: 27% 20,001–40,000: 21% 40,001–85,000: 24% ≥ 85,001: 28%	Survey completed two to six months postpartum	Marital status, prenatal participating in the Special Supplemental Nutrition Program for Women, Infants, and Children (WIC) pregnancy intention, the Kotelchuck index of prenatal care adequacy, commencement of prenatal care in the first trimester, parity, history of PTB, maternal pre-pregnancy BMI, pre-pregnancy multivitamin use frequency per week, pre-pregnancy alcoholic drink consumption, delivery method, residency, and year of delivery	PTB, LBW, SGA
Ashford et al. 2021 [33] Multisite private & academic prenatal clinics, USA 2016 to 2020	To describe ‘switching behaviours’ and associated birth outcomes among infants of women smoking cigarettes, exclusively vaping, or using both (dual use)	Prospective cohort study *n* = 218 women	Mean (SD) 27.9 (7.3) Range 18–44	White 85%	Annual household income: < $49,999 78.9% Some college education 73%	First or second trimester	History of PTB, smoking in first trimester, marital status, household income, employment status	GA, BW, neonatal intensive care unit admissions, respiratory distress
Cardenas et al. 2019 [34] Single site, University affiliated low-risk pregnancy clinic, USA 2015–2017	To explore the effect of vaping on BW and SGA	Prospective cohort study *N* = 232 women *N* = 199 when excluding those who did not disclose smoking status	18 – 22 years: 38% 23–27: 31% ≥ 28: 32%	Non-Hispanic Black 45% Non-Hispanic White 38%	High school and above 75.8%	Single assessment during pregnancy GW at assessment: GW < 20 33.9% GW > 20 65.3% Missing 0.8%	Exposure to second-hand smoke/vaping	BW, SGA
Cardenas et al. 2020 [35] State-wide survey using Pregnancy Risk Assessment Monitoring (PRAMS) data, USA 2016–2017	To estimate the effect of vaping & smoking use, and smoking cessation, during pregnancy on smallness-for-gestational-age	Retrospective cohort study, secondary analysis. Restricted to those with complete data on smoking, vaping, SGA *n* = 1,594 women	≤ 19: 52% 20–34: 51% ≥ 35: 88% Maternal age range NR	Non-Hispanic Black 55% Non-Hispanic White 48%	Some college or above 49%	Survey completed two to six months postpartum	Pre-pregnancy alcohol use	SGA
Choi 2024 [36] Pregnancy Risk Assessment Monitoring System (PRAMS) USA 2016–2019	To examine the association of vaping with PPD	Retrospective cohort study, secondary analysis. Restricted to those with complete data on smoking and vaping *N* = 58,950 women	< 20: 3.6% 20–24: 17.8% 25–29: 29.7% 30–35: 30.3% > 34: 18.5%	White 69.2% Black 19.3% Asian 4.0% American Indian & Alaskan Native 3.2% Other 4.4% Hispanic 17.1%	Annual household income $ 0–23,999: 35.2% 24,000–47,999: 19.4% 48,000–72,999: 12.9% > 73,000: 26.6%	Survey completed two to six months postpartum	Combustible cigarette, and/or hookah use, prenatal care during the last trimester, health insurance coverage during pregnancy, physical abuse during pregnancy, method of survey response	Postpartum depression with two questions related to frequency of (i) depression and hopelessness and (ii) little interest or pleasure in doing things they usually enjoy
Clemens et al. 2019 [37] Single site, University-affiliated medical centre, USA 2015–2016	To compare levels of nicotine, cotinine, and tobacco-specific nitrosamines in hair samples from pregnant women who were exclusive-smokers, dual smokers/vapers, and non-users. To estimate association between vaping/ smoking and smallness for gestational age	Prospective cohort study *N* = 76 women *N* = 58 when self-reported vaping/ smoking was consistent with hair sample	≥ 18	NR	NR	Mean (SD) 18 (0.6) GW	NR	SGA, Nicotine metabolites, tobacco specific nitrosamines (TSNA)
Cohn et al. 2023 [38] Nationally representative study (Population Assessment of Tobacco and Health (PATH)), USA 2013–2019	To investigate associations of prenatal vaping with pregnancy and birth outcomes	Prospective cohort study, secondary analysis *N* = 1037 women	18–24: 45% 25–34: 45% 35 + : 10%	Non-Hispanic white 54%	College or associates degree or higher 55%	Mean (SD) 21.4 (0.4) GW	Past 30-day alcohol use	Adverse birth outcomes: any incidence of PTB, LBW, baby with birth defects, placenta previa, placenta abruption, or pre-eclampsia
Coleman et al. 2021 [39] Nationally representative, study (Population Assessment of Tobacco and Health (PATH)), USA, 2013–2018	To examine urinary biomarkers of exposure, among pregnant and non-pregnant women	Retrospective cohort study, secondary analysis *N* = 1613: *n* = 109 pregnant women plus *n* = 1,504 non-pregnant women	18–49	NR	NR	NR	Second hand smoke exposure, past 30-day marijuana use, cigarettes per day, vaping in last month	Nicotine metabolites, tobacco specific nitrosamines (TSNA), Polycyclic aromatic hydrocarbon (PAH) metabolites, Volatile organic compound (VOC) metabolites, Heavy metals
Froggatt et al. 2020 [40] Single site, University Hospital UK Dates not reported	To assess whether birth outcomes and neuro-behavioural outcomes (in one month old infants) differed between prenatal non-exposed, smoking exposed and vaping exposed infants	Case–control study *N* = 83 infants	Infant (mean): 32.6 days Maternal (mean): Non-exposed 28.8 years; Cigarette exposed 25.5 years; vaping exposed 22.60 years; Maternal age range NR	White British 100%	College and above 55.4%	Maternal vaping/ smoking behaviour obtained at 32 GW	Additional household smokers, maternal age, infant sex, primiparous mothers, stress, depression and anxiety	GA, BW, abnormal reflexes, motor maturity, self-regulation scores, infant head circumference
Froggatt et al. 2021 [41] Two University Hospital sites, UK Dates not reported	To assess whether foetal mouth movement frequency changes across gestation and whether there are differences between cigarette and e-cigarette exposed foetuses compared with non-exposed foetuses	Prospective cohort study *N* = 123 women recruited Included in analysis: *n* = 106 women at 32-week scan *n* = 86 at 36-week scan 79 sets of paired 32- and 36-week data	18–40	NR	NR	Scans at 32 and 36 weeks gestation	Time of scan, antenatal attachment, maternal anxiety, stress, depression	Fetal mouth movements
Galbo et al. 2022 [56] National survey using Pregnancy Risk Assessment Monitoring (PRAMS) data, USA 2016–2017	To investigate the association between women who vape during the last three months of pregnancy and unfavourable birth outcomes in comparison to women who did not vape	Retrospective cohort study, secondary analysis. Restricted to those with complete data on vaping in last three months of pregnancy and for outcomes of interest *N* = 71,940 women	< 19: 5.0% 20–24: 18.9% 25–34: 58.2% > 35: 17.8%	White: 57.3% African American: 18.2% Asian: 6.7% Hispanic: 18.0% Other: 14.4%	Higher education: 61.9%	Survey completed two to six months postpartum	Insurance, maternal education, Kotelchuck index, physical abuse during pregnancy, and complications during pregnancy including gestational diabetes, high blood pressure, and clinical depression	The dependent variable was dichotomized into the presence of at least one unfavourable birth outcome (PTB, LBW, or extended postnatal hospital stay for the newborn) or the absence of all Gestational diabetes, high blood pressure, depression
Hajek et al. 2022 [16] Pesola et al. 2022 [57] 23 hospital sites UK 2018–2020	To compare efficacy and safety of vaping and NRT for smoking cessation in pregnancy A secondary analysis (Pesola et al. 2022^xx^) compared safety outcomes in participants who did and did not regularly use these two nicotine products (i.e., combined group of those vaping or using NRT) during pregnancy, regardless of randomisation	Randomised Control Trial Baseline: *n* = 1140 women Birth Outcomes: *n* = 1095	> 18 Median 27	White British 88%	At least further education 59%	Mean 15.7 GW	Secondary analysis (Pesola et al. 2022^xx^), comparing outcomes for those using nicotine products (vaping or NRT) and those not using these products was adjusted for baseline cotinine levels, occupation and Fagerstrom Test of Cigarette Dependence Analyses comparing incidence of respiratory symptoms between nicotine product users and non-users were adjusted for smoking status	GA, PTB, BW, LBW, neonatal intensive care unit admissions, terminations, miscarriages, still births, neonatal death, post-neonatal death, congenital abnormalities, caesarian-section delivery, maternal respiratory symptoms (cough, phlegm, shortness of breath, wheezing), presence of any adverse pregnancy outcome
Hawkins et al. 2021 [42] National survey using Pregnancy Risk Assessment Monitoring (PRAMS) data, USA 2016–2017	To examine the associations between vaping and smoking during pregnancy with birth outcomes	Retrospective cohort study, secondary analysis. Restricted to those with complete data on smoking, vaping and maternal age *n* = 57,046 women	Maternal age/range NR	NR	NR	Survey completed two to six months postpartum	Method of payment for delivery, use of Special Supplemental Nutrition Program for Women, Infants, and Children during pregnancy, marital status, trimester of first prenatal care visit, language preference, plurality, and parity	PTB, BW, SGA
Kim et al. 2020 [43] National survey using Pregnancy Risk Assessment Monitoring (PRAMS) data, USA 2016–2018	To examine the effects of prenatal vaping on neonatal birth outcomes compared with smoking and non-use of tobacco	Retrospective cohort study, secondary analysis. Restricted to those with complete data on smoking, vaping, outcomes of interest, covariates *N* = 55,251 Final analysis *N* = 53,971 women	Median (IQR): 27 (22–32) Maternal age range NR	White 48%	Some college or above 64% Annual household income ($): < 25,000: 38.2% 25,000–50,000: 20.2% 50,000–75,000: 13.3% > 75,000 28.4%	Survey completed two to six months postpartum	Adequate prenatal care, yearly income	PTB, LBW, SGA
Lavezzi et al. 2022 [44] Hospital setting, Sites and dates not reported, Italy	To evaluate in-depth histopathological examinations of the autonomic nervous system in those with sudden intrauterine unexplained deaths, with and without lung hypoplasia, and controls, and in relation to smoking and vaping status	Case control study *N* = 67	< 20 years: 45% ≥ 20 years: 55% Maternal age range NR	White 73%	Normal/high socioeconomic status 48%	25–40 GW	Infant gender, maternal obesity, alcohol and drug use, exposure to airborne particulate matter, pesticides, solvents, other toxicants	Adverse neuropathological brainstem outcomes: pulmonary hypoplasia, and pFn hypoplasia
Lin et al. 2023 [45] Nationally representative study (Population Assessment of Tobacco and Health (PATH)), USA, 2011–2018	To evaluate the association between vaping before and during pregnancy and adverse birth outcomes and explore the association between vape flavourings and adverse birth outcomes	Retrospective cohort study, secondary analysis *N* = 597 women High risk birth *n* = 75 Foetus death *n* = 34 Normal birth *n* = 488 Substantial loss of data included in adjusted analyses for the following analyses: Vaping and high-risk birth *n* = 375 (63% of full sample of 597); Vaping and foetus death *n* = 402 (67%); Vaping flavours and high-risk birth *n* = 76 (13%); vaping flavours and foetus death *n* = 132 (22%)	18 to 44	NR	NR	NR	Education, ethnicity, maternal age, satisfaction & social activities & relationships. perception of vaping compared with other tobacco products; whether received advice from a healthcare professional to quit tobacco/e-cigarettes within the prior 12 months, overall health status, cigarette, alcohol, and marijuana use prior to and during pregnancy, and whether smoking allowed at home	High-risk birth, defined as any incidence of PTB, LBW, baby birth defects, placenta previa, placenta abruption, pre-eclampsia, & cleft lip or palate Fetal death
McDonnell et al. 2019 [46] Single sites, urban maternity hospital, Ireland 2017–2018	To compare obstetric outcomes and socio-demographic factors in vapers cigarette smokers and non-smokers/vapers in pregnancy	Prospective cohort study *N* = 620 women	Mean 30.5 Maternal age range NR	Irish 84%	Professional/ managerial 24%	10–14 GW	Parity, planned pregnancy, pre-conception folic acid, height, weight, body mass index	BW, LBW, MBC, neonatal Intensive Care Unit (NICU) admissions, APGAR scores
Nanninga et al. 2023 [47] National survey in Netherlands 2020–22	To investigate pregnancy outcomes according to maternal smoking and vaping status	Retrospective cohort study (reported in publication as a cross-sectional study) *N* = 1,937 women	Mean age 30.5 Range: 17 to 44 years	NR	48% attended higher education	Up to 12 months postpartum	NR	PTB, SGA, hospital admissions, composite infant outcome, at least one of: PTB, SGA, hospital admission, or stillbirth. Pregnancy outcomes: hypertensive disorders gestational diabetes postpartum haemorrhage, miscarriage, composite adverse pregnancy outcome
Opondo et al. 2021 [48] National Maternity (postal) Survey, UK 2018	To explore associations between maternal characteristics and vaping, and between vaping and birth outcomes	Retrospective cohort study (reported in publication as ‘a cross-sectional population-based survey’) Sample for analysis: *n* = 4,421 women	Mean age 31 years Age 16 years or older	Weighted: White British 67%	Weighted: Index of multiple deprivation 1&2 49%	6 months postpartum	Parity, planned pregnancy, reaction to pregnancy, living with smoker	PTB, BW
Regan, Bombard et al. 2021 [49] National survey using Pregnancy Risk Assessment Monitoring (PRAMS) data, USA 2016–2018	To evaluate the risk of adverse birth outcomes among vapers before and during pregnancy and to assess whether this association varied by frequency of vaping	Retrospective cohort study, secondary analysis. Restricted to infants with birth weights of 400 g or higher, complete data on smoking, vaping and covariates *n* = 79,176 women	Weighted: 18–24: 22% 25–29: 30% 30–34: 30% 35–39: 15% ≥ 40: 0.03%	Weighted: White Hispanic 61%	Weighted: Education ≥ 16 years 37%	Survey completed two to six months postpartum	Use of Special Supplemental Nutrition Program for Women, Infants and Children services during pregnancy, adequacy of prenatal care, multivitamin use, combustible cigarette use during pregnancy	PTB, LBW, SGA
Regan & Pereira 2021 [50] National survey using Pregnancy Risk Assessment Monitoring (PRAMS) data, USA 2016–2018	To describe vaping patterns among women who smoked pre-pregnancy and assess whether vaping during pregnancy was associated with adverse birth outcomes in comparison to continued smoking	Retrospective cohort study, secondary analysis. Restricted to those reporting smoking in the 2 years prior to pregnancy and to infants with birth weights of 400 g or higher and complete data on smoking, vaping and covariates *n* = 16,022 women	18–24: 31% 25–29: 33% 30–34: 24% 35–39: 10% ≥ 40: 0.2%	White Hispanic 72%	Education ≥ 16 years 14%	Survey completed two to six months postpartum	Parity, adequacy of prenatal care, multivitamin use, presence of obstetric risk factor	PTB, LBW, SGA
Rollins et al. 2020 [51] Single site, low-income urban clinic, USA 2015–2018	To assess depressive symptoms in pregnant women who vaped during pre-conception and/or pregnancy	Retrospective cohort study *N* = 1365 women	Mean (SD) 27.1 (5.2) Range 16–45	White Hispanic 36% Hispanic/Latin 25%	College or Higher 15%	Mean (SD) 13.7 (9.2) GW	Alcohol use, Marijuana/ other substance use in pregnancy and preconception	Depressive symptoms
Shittu et al. 2022 [52] National survey using Pregnancy Risk Assessment Monitoring (PRAMS) data, USA 2016–2018	To characterise changes in vaping and smoking from before to during pregnancy and examine their associations with SGA	Retrospective cohort study, secondary analysis. Restricted to those with data on smoking, vaping and SGA *N* = 105,438 women	Weighted sample: ≤ 19: 5% 20–24: 19% 25–29: 29% ≥ 30: 48% Maternal age range NR	Weighted: White Non-Hispanic 58%	Weighted: Some college or higher 63% Annual household income $ (weighted): < 24,000: 33.7% 24,001–48,000: 19.7% 48,001–85,000: 19.0% > 85,000: 27.5%	Survey completed two to six months postpartum	Type of health insurance, pre-pregnancy body mass index	SGA
Wang et al. 2020 [53] National survey using Pregnancy Risk Assessment Monitoring (PRAMS) data, USA 2016	To examine patterns of vaping before and during pregnancy and to evaluate the association of vaping with PTB and foetal growth restriction	Retrospective cohort study, secondary analysis. Restricted to those with complete information on smoking and vaping *N* = 31,973 women	Weighted %: Weighted %: < 20 years: 5% 20–24: 18% 25–29: 29% 30–34: 30% 35 + : 18% Maternal age range NR	Weighted: Non-Hispanic White 52%	Weighted: Some college or higher 64%	Survey completed two to six months postpartum	Marital status, parity, previous preterm delivery, adequacy of prenatal care, maternal pre-pregnancy body mass index, pre-pregnancy alcohol consumption, gestational weight gain	PTB, SGA
Wang et al. 2022 [54] National survey using Pregnancy Risk Assessment Monitoring (PRAMS) data, USA 2016–2018	To investigate the effect of vaping during pregnancy on neonatal outcomes with a primary aim of assessing exposure–response associations while accounting for concurrent smoking and vaping	Retrospective cohort study, secondary analysis. Restricted to those with complete information on smoking and vaping *N* = 99,201 women	< 20 years: 5% 20–24: 19% 25–29: 29% 30–34: 29% 35 + : 18% Maternal age range NR	Non-Hispanic White 46% Non-Hispanic Black 18% Hispanic 20% Other 16%	Some college or higher 62%	Survey completed two to six months postpartum	Birth year, mother’s marital status, parity, previous preterm delivery, adequacy of prenatal care, drinking alcohol before pregnancy and pre-pregnancy BMI, smoking and vaping in the three months before pregnancy	PTB, SGA
Wen et al. 2023 [55] National survey using Pregnancy Risk Assessment Monitoring (PRAMS) data, USA 2016–2020	To evaluate low gestational weight gain in women who exclusively vape, exclusively smoke, dual use or do not smoke or vape	Retrospective cohort study, secondary analysis. Restricted to those with data on vaping and smoking in last 3 months of pregnancy, pre-pregnancy BMI, and total gestational weight gain *N* = 176, 882 women	< 19 years: 5% 20–24: 19% 25–29: 29% > 30: 48% Maternal age range NR	White 59%	Some college or higher 65%	Survey completed two to six months postpartum	Maternal age education, ethnicity, marital status, type of health insurance, pre-pregnancy hypertension pre-pregnancy diabetes, pre-pregnancy body mass index	Gestational weight gain

*NR* not reported, *SD* standard deviation, *BMI* body mass index, *PTB* pre-term birth, *GA* gestational age, *BW* birth weight, *LBW* low birth weight, *SGA* small for gestational age

**Table 2:** Vaping and smoking groups and associated characteristics

Author/date	Study groups, definition: % (n)	Biochemical validation	Dependence measures	Other smoking and vaping characteristics reported ^a^	Second-hand exposure
Ammar et al. 2023 [32]	*Reported in the last three months of pregnancy:* Exclusive-vapers: 0.5% (977/190,707) Exclusive-smokers: 7.7% (14,752/190,707) Dual-users: 0.7% (1,404/190,707) Non-users: 91.0% (173,574/190,707) *Among those reporting any vaping in the three months before pregnancy (N* = *7,877):* Reported any vaping in last three months of pregnancy 25.4% (2,002/7,877) Reported no vaping in last three months of pregnancy 74.6% (5,875/7,877)	NR	NR	Frequency of vaping during the last 3 months of pregnancy among those who vaped in the 3 months before pregnancy: None: 74.6% (5,875/7,877) < 1 day/week: 9.5% (747/7,877) 2–6 days/week: 3.6% (280/7,877) Once per day: 3.7% (289/7,877) > 1per day: 8.7% (686/7,877)	NR
Ashford et al. 2021 [33]	*At enrolment, self-report with nicotine level verified:* Exclusive-vapers: 10.0% (21/218) Exclusive-smokers: 48.6% (106/218) Dual-users: 41.7% (91/218) *Change of product use over pregnancy:* Switched products: 26.1% (57/218) Quit both: 11.5% (25/218) Continued use as at enrolment: 62.4% (136/ 218), comprising: 5.8% (8/136) vapers, 49.2% (67/136) smokers, 33% (45/136) dual-users	At enrolment, urine cotinine used to assess smoking status. Serum cotinine measured at each study visit	NR	NR	NR
Cardenas et al. 2019 [34]	*Use in past month:* Exclusive-vapers: 3.0% (6/199) Exclusive-smokers: 28.1% (56/199) Dual-users: 8.5% (17/199) Non-users (validated as non-users): 32.2% (64/199) Other tobacco use: 5.5% (11/199)	Salivary cotinine and hair nicotine used to assess smoking status	NR	Frequency: Vaping daily to 10 days a month 20.8% (5/24), 3 to 9 times a month 29.2% (7/24), 1 to 2 times a month 50% (12/24) Smoking: Dual-users: mean 6.3 cigarettes per day, exclusive smokers: mean 7.3 cigarettes per day Use of other tobacco products was assessed	NR
Cardenas et al. 2020 [35]	*Use in 3 months before pregnancy or in last 3 months of pregnancy:* Exclusive-vapers: 1% (18/1594) Exclusive-smokers: 23% (372/1594) Dual-users: 6% (100/1594) Non-users: 67% (1062/1594) Other tobacco use: 3% (42/1594)	Sensitivity analysis carried out using sensitivity and specificity estimates from previous validation studies using hair nicotine and serum cotinine measures	NR	NR	NR
Choi et al. 2024 [36]	*Numbers are unweighted while percentages are weighted* *Reports of vaping in the past two years:* Any vaping 6.6% (3,874) No vaping 93.4% (55,076) Reports of vaping in the 3 months before pregnancy (among those who vaped in the past two years): Any vaping 58.1% (2,241) No vaping 41.9% (1,638) Reports of vaping in the last 3 months of pregnancy (among those who vaped in the past two years): Any vaping 18.2% (703) No vaping 81.8% (3,193) *Reports of use of* combustible cigarettes and/or hookah *in the past two years:* 20.9% (12,323)	NR	NR	*Numbers are unweighted while percentages are weighted* Frequency of vaping during the 3 months before pregnancy: > Once a day: 763 (21.9) Once a day: 204 (4.6) 2–6 days a week: 289 (7.5) < 1 day a week: 985 (24.9) *Frequency of vaping during the last 3 months of pregnancy:* > Once a day: 226 (6.8) Once a day: 106 (2.1) 2–6 days a week: 103 (2.5) < 1 day a week: 268 (6.8)	NR
Clemens et al. 2019 [37]	*Use in past month:* Exclusive-smokers: 41.3% (24/58) Dual-users: 15.5% (9/58) Non-users: 43.1% (25/58)	Hair nicotine and cotinine levels	NR	NR	NR
Cohn et al. 2023 [38]	*Use in past 30-days:* Vapers/dual-users: 6.7% (69/1037) Exclusive-smokers: 22.8% (236/1037) Non-users: 70.6% (732/1037)	NR	Fagerstrom Test Waking First Cigarette Score: ≤ 5 min: 5%; 6–30 min: 4%; 31–60 min: 7%; > 60 min: 6%	NR	NR
Coleman et al. 2021 [39]	*Use some days or every day at time of survey:* *Pregnant (n* = *109):* Exclusive-vapers: 6% (7/109) Exclusive-smokers: 77% (84/109) Dual-users: 17% (18/109) *Non-pregnant* (*n* = 1504): Exclusive-vapers: 7% (111/1504) Exclusive-smokers: 68% (1023/1504) Dual-users: 25% (370/1504)	NR	NR	*Number of days of vaping past month (95% CI):* Pregnant Vapers: 10.63 (3.41 to 17.85) Dual-users: 5.09 (0.80 to 9.39) Non-pregnant Vapers: 17.13 (13.62 to 20.65) Dual-users: 7.45 (5.09 to 9.80) *CPD (95% CI):* Pregnant Dual-users: 8.75 (5.26 to 12.24) Smokers: 9.80 (7.18 to 12.42) Non-pregnant Dual-users: 9.44 (7.88 to 11.00) Smokers: 9.48 (7.33 to 11.36)	NR
Froggatt et al. 2020 [40]	*Use at time of survey (32 weeks gestation):* Exclusive-vapers: 12% (10/83) Exclusive-smokers, 1 to 20 CPD: 35% (29/83) Non-users, no exposure to nicotine/tobacco products: 53% (44/83)	Expired carbon monoxide level	NR	Pre-conception mean years smoking: Vapers: 4.2 Smokers: 11.2 Non-users: 0.3 Vaping nicotine dose: 3–16 mg Women using NRT or other nicotine product were ineligible	Additional household smokers: Vapers: 2.4% (2/83) Smokers: 12% (10/ 83) Non-users: 3.6% (3/83)
Froggatt et al. 2021 [41]	*32 weeks gestation:* Exclusive-vapers: 14.1% (15/106) Exclusive-smokers, < 10 CPD: 32 weeks: 30.1% (32/106) Exclusive-smokers, 11–20 CPD: 32 weeks: 12.2% (13/106) Non-users: 43.3% (46/106) *36 weeks gestation:* Exclusive-vapers: 16% (14/87) Exclusive-smokers, < 10 CPD: 31% (27/87) Exclusive-smokers, 11–20 CPD: 13.7% (12/87) Non-users: 39.1 (34/87)	Expired carbon monoxide level	NR	Vaping nicotine dose: 3–16 mg Mean (SD) = 7.8 (4.8) mg Heavy smokers (11–20 CPD day) 28% Use of other nicotine products assessed but not reported	NR
Galbo et al. 2022 [56]	*Reported any vaping in the last three months of* pregnancy: 1.2% (859/71,940)	NR	NR	NR	NR
Hajek et al. 2022^15^ Pesola et al. 2024 [57]	*At enrolment* Smokers: 100% (1140) 50% randomised to NRT, 50% to vaping *Secondary analysis of groups who did and did not regularly use vaping or NRT in pregnancy, regardless of randomisation (*Pesola et al. 2024 [57]: Regular vaping during pregnancy (*N* = 539, 47.3) Regular use of NRT (*N* = 235, 20.6%). Among abstainers, there were 131 (66.8%) regular users of vaping and 40 (20.4%) regular users of NRT, 25 (12.8%) abstainers did not use any nicotine products regularly	Salivary cotinine, salivary anabasine & expired carbon monoxide level	Fagerstrom test of cigarette dependence score, mean (SD) Vaping: 4.0 (2.1) NRT: 4.3 (2.1)	Median 10 CPD at baseline Treatment adherence: Vaping: 77% (438/571) NRT: 51% (292/569) Refillable e-cigarettes 94%, maximum 20 mg/ml nicotine, nicotine concentration decreased significantly over time; most used tobacco and fruit flavours Trial intervention period from enrolment to end of pregnancy NRT: 17.8% vaped regularly Vaping: 2.8% regularly used NRT Regular use of vaping or NRT was defined as self-report of having used the product for at least 5 consecutive days during the first 4 weeks, using regularly for at least 1 week, or occasionally for at least 3 weeks or currently using the product at EOP	Lives with person who smokes: Vaping: 59.9% NRT: 57.6%
Hawkins et al. 2021 [42]	*Last 3 months of pregnancy:* Exclusive-vapers: 0.5% (301/57,046) Exclusive-smokers: 8% (5063/57,046) Dual-users: 0.8% (529/57,046) Non-users: 89.7% (51,153/57,046)	NR	NR	NR	NR
Kim et al. 2020 [43]	*Last 3 months of pregnancy:* Exclusive-vapers: 0.6% (337/55251) Exclusive-smokers: 6% (3484/55251) Non-users: 93% (51,340/ 55,251)	NR	NR	NR	NR
Lavezzi et al. 2022 [44]	*Period of assessment not stated:* *Sudden intrauterine unexplained deaths (SIUDS), lung hypoplasia group (n* = *25):* Exclusive-vapers: 20% (5/25) Exclusive-smokers, > 3 CPD: 60% (15/25) Dual-users: 16% (4/25) Non-users: 4% (1/25) *SIUDS normal lung development group (n* = *22):* Exclusive-smokers: 45% (10/22) Non-users: 55% (12/22) *Well defined death (Control Group) (n* = *20):* Exclusive-smokers: 20% (4/20) Non-users: 80% (16/20)	Hair cotinine	NR	NR	NR
Lin et al. 2023 [45]	Compared four vaping patterns in pregnancy (size of groups NR): Continuous exclusive-vapers/dual-users The other three groups included both non-vapers/non-smokers and smokers: Stopped vaping before pregnancy Stopped vaping in pregnancy Never vaped *Also compared vaping flavours groups:* Menthol/mint Alcohol Candy Other Size of groups NR	NR	NR	Assessed use of vaping flavours, classed as mint/menthol, alcohol, candy, and others, but didn’t report frequencies	Controlled for whether smoking is allowed in the home
McDonnell et al. 2019 [46]	*Use at time of survey (10 to 14 weeks gestation):* Exclusive-vapers: 35% (218/620) Exclusive-smokers, at least 1 CPD: 16% (99/620) Dual-users: 31% (195/620) Non-users, never used: 17% (108/620)	NR	NR	NR	NR
Nanninga et al. 2023 [47]	Covers whole of pregnancy, assessed postpartum: Exclusive-vapers 0.5% (10/1937) Exclusive-smokers 10.8% (209/1937) Dual-users 0.6% (12/1937) Non-users 88.1% (1706/1937)	NR	NR	*Use throughout pregnancy:* Vapers 50% Smokers 52% Dual-users 58% *Use for* > *3 years before pregnancy:* Vapers 40% Smokers 86% Dual-users 58% 3 vapers used non-nicotine devices, 7 used a low nicotine dose. 3 dual-users used non-nicotine vapes, 6 used a low nicotine dose and three used a high dose	
Opondo et al. 2021 [48]	*After finding out about pregnancy* Exclusive-vapers: 0.6% (27/4,421) Exclusive-smokers: 4.1% (183/4,421) Dual-users: 1.6% (71/4,421) Non-users: 93.6% (4,139/4,421) Unknown: *n* = 1	NR	NR	*Smoking frequency:* ‘daily’, ‘less than daily but at least once a week’, ‘less than weekly but at least once a month’, ‘less than monthly’ and ‘not at all’ *Four categories of smokers:* ‘Ex-smokers’ 17.6%: previously smoked but mostly not smoking at all ‘Temporary quitters’ 4.0%: smoked almost daily before pregnancy but reduced frequency of smoking, resuming original frequency after the birth ‘Pregnancy-inspired quitters 6.2%: maintained reduced frequency of smoking after birth ‘Persistent smokers’ 6.8%: continued smoking daily at similar frequency as before pregnancy	NR
Regan, Bombard et al. 2021 [49]	*During last 3 months of pregnancy:* Exclusive-vapers: 0.4% (329/79,176) Exclusive-smokers: 6.4% (5,100/79,176) Dual-users: 0.7% (577/79,176) Non-users: 89.7% (71,013/79,176) *During the 3 months before pregnancy:* Exclusive-vapers: 0.6% (462/79,176) Exclusive-smokers: 16.4% (13,015/79,176) Dual-users: 2.1% (1695/79,176) Non-users: 79.7% (63,098/79,176)			*Frequency of vaping* 3 months before pregnancy: 39.8% daily vapers Last 3 months of pregnancy – 44.3% daily vapers	
Regan & Pereira 2021 [50]	*During the last three months of pregnancy* Exclusive-vapers: 1% (189/16,022) Exclusive-smokers: 39% (6310/16,022) Dual-users: 4% (585/16,022) Non-users, all former smokers: 56% (8938/ 16,022)	NR	NR	*Frequency of vaping* 3 months before pregnancy: 36.3% daily vapers Last 3 months of pregnancy – 61.3% daily vapers	NR
Rollins et al. 2020 [51]	*Current use and/or use 3 months preconception:* Exclusive-vapers: 1% (14/1365) Exclusive-smokers: 27% (372/1365) Dual-users: 3% (40/1365) Non-users: 69% (939/1365)	NR	NR	Mean CPD: Dual-users: 8.9 Smokers: 6.2	NR
Shittu et al. 2022 [52]	*In 3-months pre-pregnancy,* (*weighted %*) Exclusive-vapers: 1% (61,173/5,446,900) Exclusive-smokers: 14% (773,586/5,446,900) Dual-users: 3% (149,152/5,446,900) Non-users: 82% (4,462,989/5,446,900) *Changes during pregnancy* *Vapers:* Quit vaping: 80.7% (49,367/ 61,173) Continued vaping: 18.6% (113,78/ 61,173) Switched from vaping to smoking: 0.63% (385/61,173) Continued vaping and started smoking: 0.01% (6/61,173) *Smokers:* Quit smoking: 54.4% (420,830/773,586) Continued smoking: 44.1% (317,944/773,586) Switched from smoking to vaping: 0.46% (3558/773,586) Continued smoking and started vaping (dual use): 0.97% (7504/773,586) *Dual-users:* Quit smoking & vaping: 46.4% (69,207/149,152) Quit vaping & continued smoking: 26.7% (39,824/ 149,152) Quit smoking and continued vaping: 5.6% (8353/ 149,152) Continued dual use 21.4% (31,919/149,152) Non-users: Started vaping and smoking: 0 Started vaping: 0.07% (104/149,152) Started smoking: 0.07% (104/149,152) Continuous non-user: 99.9% (148,994/149,152)	NR	NR	NR	NR
Wang et al. 2020 [53]	*In 3-months pre-pregnancy* Exclusive-apers: 1% (267/31,973) Exclusive-mokers: 16% (5029/31,973) Dual-users: 3% (976/31,973) Non-users: 80% (25,521/31,793) *Changes during last 3 months of pregnancy* *Smokers:* Quit smoking: 52% (2622/5,029) Continued smoking: 47% (2342/5,029) Switched to vaping: 0.3% (18/5,029) Switched to dual use: 0.9% (47/5,029) Vapers: Quit vaping: 81% (215/267) Continued vaping: 18% (49/267) Switched to smoking: 0.1% (3/267) Switched to dual use: 0% Dual-users: Quit smoking & vaping: 44% (432/976) Quit vaping, continued smoking: 28% (270/976) Quit smoking, continued vaping: 6% (56/976) Continued dual use: 22% (218/976) Non-users: Continued non-use: 99% (25,501/25,521) Started to smoke 0.06% (17/25,521) Started to vape 0.01% (3/25,521) Started dual use: 0	NR	NR	NR	NR
Wang et al. 2022 [54]	*Reported in the last three months of pregnancy:* *(unweighted N, Weighted %)* Exclusive-smokers: 7.1% (8164) Exclusive-vapers: 0.4% (406) Dual users: 0.7% (723) Non-users: 91.8% (89,908) All categories *in the last three months of pregnancy: (unweighted N, Weighted %)* 1. Non-users 91.8% (89,908) 2. Exclusive occasional vapers (vaping < once a day) 0.2% (176) 3. Exclusive frequent vapers (vaping ≥ once a day) 0.2% (230) 4. Dual-users: light smoker (1–5 cigs/day) & occasional vaper 0.2% (230) 5. Dual users: light smoker & frequent vaper 0.1% (163) 6. Exclusive light smoker: 4.2% (4,988) 7. Dual-users: heavy smoker (≥ 6 cigs/day) & occasional vaper 0.2% (212) 8. Dual users: heavy smoker and frequent vaper 0.1% (118) 9. Exclusive heavy smoker 2.9% (3176)	NR	NR	Frequency of vaping during the three months before pregnancy, and during the last three months of pregnancy Daily average quantities of cigarettes smoked was also queried for those two time periods. (see study groups column for %)	NR
Wen et al. 2023 [55]	*Any use in last 3 months of pregnancy* Exclusive-vapers: 0.5% (921/176,882) Exclusive-smokers: 7.7% (13,697/176,882) Dual-users: 0.7% (1,308/176,882) Non-users: 91.0% (160, 956/176,882)	NR	NR	NR	NR

*NR* not reported, *CPD* cigarettes per day, *NRT* nicotine replacement therapy

^a^Frequency and duration of vaping/smoking, product type/dose/ flavours, use of other nicotine or tobacco products

#### Study design

One RCT (comparing vaping and NRT for smoking cessation in pregnancy, *n* = 1,095) [16], reported outcomes by randomisation group and when combining the whole sample who were using either vaping or NRT, irrespective of randomisation [57]. We did not count this latter secondary analysis as a separate study as it analysed the same data as the main study. There were 23 cohort studies, including 17 retrospective studies (i.e., use of nicotine and tobacco products was assessed retrospectively [32, 35, 36, 39, 42, 43, 45, 47–56] and six prospective studies (i.e., product use assessed across time) [33, 34, 37, 38, 46]. Fifteen cohort studies involved secondary data analysis of US data, including twelve from Pregnancy Risk Assessment Monitoring (PRAMS) data [32, 35, 36, 42, 43, 49, 50, 52–56] and three from the Population Assessment of Tobacco and Health (PATH) [38, 39, 45]. We counted these secondary analyses as separate studies as, in cases where they assessed the same outcome, each study used a different set of data, with different sample sizes and with the studies varying not only in the years sampled (range: PRAMS 2016–2020, PATH 2011–2019) but also in inclusion criteria (e.g., two PRAMS studies restricted the data to infants with birth weights of 400g or higher) and in the covariates used. There were also two case–control studies [40, 44].

#### Study location and setting

Nineteen studies were conducted in the US [32–39, 42, 43, 45, 49–56], four in the UK [16, 40, 41, 48], one in Italy [44], one in Ireland [46] and one in the Netherlands [47]. Five studies were conducted at single-sites [34, 37, 40, 46, 51], three at multiple-sites [16, 33, 41], one was a US Statewide survey [35], 16 were national surveys [32, 36, 38, 39, 42, 43, 45, 47–50, 52–56] and one study did not report the setting [44].

#### Maternal characteristics

Ethnicity and/or race was reported in all but six studies [37, 39, 41, 42, 45, 47]; 16 reported predominantly white ethnicity [16, 32, 33, 36, 38, 40, 44, 46, 48–50, 52–56]. Maternal age was reported by all but one study [42] but using ranges, means, or categories, making summaries difficult. Socioeconomic status/education/income was reported in all but five studies [37, 39, 41, 42, 45]; most reported predominantly low socioeconomic status/education/income. Gestational age/trimester or infant age at the time of enrolment or data collection was reported in all but two studies [39, 45], ranging from 10 weeks pregnant [46] to 12 months postpartum [47], with the majority of studies interviewing women in the postpartum period.

#### Vaping and smoking groups and characteristics (see Table 2)

##### Comparisons

Nine studies compared outcomes for all four groups key groups: exclusive-smokers, exclusive-vapers, dual-users and non-users [32, 34, 35, 42, 44, 46, 47, 53, 55]. Fifteen studies compared exclusive-vaping and non-use of nicotine or tobacco products [34, 35, 40–44, 46, 47, 49, 50, 52–55] and eight studies compared exclusive-vaping and exclusive-smoking [39, 41, 43, 44, 46, 47, 50, 52]. The RCT initially compared safety outcomes for those allocated to the vaping and NRT arms [16] and then, in a secondary analysis, compared outcomes for those who were not smoking and who did or did not regularly use nicotine products (i.e., vaping or NRT, combined) during pregnancy [57]. Four studies compared outcomes for those switching products [32, 33, 35, 52].

##### Verification

One study (the RCT) biochemically verified smoking and vaping status [16], six verified only smoking status (33,34,37,40,41,44] and the others had no verification. Three studies considered the potential confounding effect of second-hand smoke exposure [16, 40, 45] and two included a broad assessment of the use of nicotine products, including NRT [16, 40]. To illustrate the importance of biochemical verification and comprehensive assessment of nicotine product usage, one study revealed that 51% of those reporting being non-users, and not having exposure to second-hand smoke or vaping, were subsequently found to have salivary cotinine or CO levels compatible with tobacco use [34]. One study stated that those counted as vaping included those using non-nicotine vapes [47], and in 17 studies it was not assessed whether any of those counted as vaping used non-nicotine vapes [32, 35, 36, 38, 42, 43, 45, 46, 48–56].

##### Vaping or smoking characteristics

Four studies reported the strength of nicotine in the vaping product [16, 40, 41, 47] three reported the duration of vaping and smoking [16, 40, 47], five reported the frequency of vaping and smoking [16, 34, 39, 50, 54], three just frequency of vaping [32, 36] three just frequency of smoking and /or cigarette consumption [41, 48, 51] and two studies assessed e-liquid flavours [16, 45]. Two studies examined the effects of the frequency of vaping on adverse outcomes [49, 54]. Three studies considered the potential confounding effect of second-hand smoke exposure [16, 40, 45] and two studies included a broad assessment of use of nicotine products, including NRT [16, 40].

##### Samples sizes

Sample sizes were often small. Of 20 studies including pregnant individuals who were exclusive-vapers, three had fewer than 10 participants in that group [34, 39, 44] seven included 10 to 30 [33, 35, 40, 41, 47, 51], nine included larger samples [32, 42, 43, 46, 49, 50, 53–55] and two did not report the sample size [45, 52]. The largest sample size of exclusive-vapers was 977 [32]. Among the 20 studies including pregnant individuals who were dual-users, two had fewer than 10 participants in that group [37, 44], three had 10 to 30 participants [34, 39, 47], 13 included larger samples [32, 33, 35, 38, 42, 46, 48–51, 53–55] and two did not report the size [45, 52]. The largest sample of dual-users was 1,404 [32].

### Quality assessment

We rated the 23 cohort studies and two case–control studies against the MMAT criteria for quantitative non-randomised studies (Table 3). Overall quality was poor. All the studies were scored as zero for the criterion ‘Were measurements appropriate regarding both the outcome and intervention (or exposure)?’ and were scored as ‘unclear’ for ‘During the study period, was the intervention administered (or exposure occurred) as intended?’. This is because none of the studies adequately measured exposure to nicotine and tobacco products; for example, through only measuring product use in late pregnancy or ‘recently’ and through not using biochemical verification. Eleven studies were considered to have unrepresentative samples. For the criterion ‘Were appropriate confounders accounted for in the design and analysis?’, three studies included all vapers without controlling for smoking status and therefore were rated as zero [38, 51, 56] as smoking is likely to be the most important confounder. All studies were considered adequate for levels of missing data. One study was rated against the MMAT RCT criteria [16] and considered to have met all criteria except for intervention adherence. This was because of contamination; among participants reporting smoking abstinence, six of 39 in the vaping arm regularly used NRT and all 25 in the NRT arm regularly vaped. Other study limitations are referred to in the results section and/or summarised and commented on in the discussion section.

**Table 3:** Quality assessment using Mixed Methods Appraisal Tool (MMAT) [30]

Study design	Randomised controlled trials criteria^a^					Quantitative non-randomised criteria^b^				
**Study**	2.1	2.2	2.3	2.4	2.5	3.1	3.2	3.3	3.4	3.5
Ammar 2023 [32]						1	0	1	1	C
Ashford 2021 [33]						0	0	1	1	C
Cardenas 2019 [34]						0	0	1	1	C
Cardenas 2020 [35]						0	0	1	1	C
Choi 2024 [36]						1	0	1	1	C
Clemens 2019 [37]						0	0	1	1	C
Cohn 2023 [38]						0	0	1	0	C
Coleman 2021 [39]						0	0	1	1	C
Froggatt 2020 [40]						0	0	1	1	C
Froggatt 2021 [41]						0	0	1	1	C
Galbo 2022 [56]						1	0	1	0	C
Hajek 2022 [16]^c^	1	1	1	1	C					
Hawkins 2022 [42]						1	0	1	1	C
Kim 2020 [43]						0	0	1	1	C
Lavezzi 2022 [44]						0	0	1	1	C
Lin 2023 [45]						1	0	1	1	C
McDonnell 2019 [46]						1	0	1	1	C
Nanninga 2023 [47]						1	0	1	1	C
Opondo 2021 [48]						1	0	1	1	C
Regan, Bombard 2021 [49]						1	0	1	1	C
Regan, Pereira 2021 [50]						1	0	1	1	C
Rollins 2020 [51]						0	0	1	0	C
Shittu 2021 [52]						1	0	1	1	C
Wang 2020 [53]						1	0	1	1	C
Wang 2022 [54]						1	0	1	1	C
Wen 2023 [55]						1	0	1	1	C

1 = criterion met, 0 = criterion not met, C = Cannot tell if criterion has been met

^a^**Randomised controlled trial criteria**

2.1 Was randomisation appropriately performed?

2.2 Were the groups comparable at baseline?

2.3 Were there complete outcome data?

2.4 Were outcome assessors blinded to the intervention provided?

2.5 Did the participants adhere to the assigned intervention?

^b^**Quantitative non-randomised criteria**

3.1 Were the participants representative of the target population?

3.2 Were measurements appropriate regarding both the outcome and intervention (or exposure)?

3.3 Were there complete outcome data?

3.4 Were apropriate confounders accounted for in the design and analysis?

3.5 During the study period, was the intervention administered (or exposure occurred) as intended?

^c^Secondary analysis of Hajek et al. 2022 [16], reported by Pesola and colleagues [57] was not separately assessed for quality

### Maternal outcomes

#### Pregnancy and postpartum outcomes

Six studies examined specific maternal pregnancy or postpartum outcomes [16,36,47,51,55,56, see Table 4]. In addition, two studies included pregnancy outcomes in a composite measure [38, 45] (see ‘Other fetal and infant outcomes’).

**Table 4:** Findings for pregnancy and postpartum outcomes

Author, date	Maternal health	Miscarriage, stillbirth, neonatal death	Other outcomes
Hajek et al. 2022 [16] Pesola et al. 2024 [57]	Vaping arm compared with NRT arm: No difference for maternal death (Relative Risk not calculated) Vapers compared with non-users (adjusted for smoking status): Significantly more likely to report improvements in cough (33.3%, 13.8%, respectively; 0.59 [0.37 to 0.93]) and phlegm (38.5%, 15.0%, respectively, 0.53 [0.31 to 0.92]); no differences in the improvement of shortness of breath (9.3%, 7.5%, respectively; 1.00 [0.62 to 1.61]) or wheezing (10.0%, 9.1%, respectively; 0.64 [0.35 to 1.16])	Vaping arm compared with NRT arm: No significant differences for miscarriage (Relative Risk (RR) [95% CIs]: 0.67 [0.11–4.00]), stillbirth (RR not calculated), neonatal death (RR 0.67 [0.11–4.00]) or postneonatal death (not calculated)	Vaping arm compared with NRT arm: No significant differences for caesarean section (RR [95% CIs]: 0.89 [0.73–1.09]) Comparisons irrespective of trial arm: Those abstinent from smoking and regularly vaping or using NRT compared with those abstinent from smoking and not using these products: Non-users had significantly more overall adverse outcomes than users (36% vs 15.7%, respectively, RR [95% CI]: 0.45 [0.24 to 0.84]) No significant differences for caesarean section rates (36.0%, 27.1%, respectively; 0.72 [0.41 to 1.29]) Those smoking and vaping or using NRT compared with exclusive-smokers: No significant difference in rates of overall adverse outcomes (4.5%, 3.1%, respectively, 1.38 [0.66 to 2.87]) No significant difference in caesarean rates (24.7%, 25.2%, respectively; 0.91 [0.71 to 1.16]) In the complete sample (combining abstainers and smokers), nicotine product users (i.e., vaping or NRT) did not differ significantly from non-users in serious adverse events (12.1% vs 14.7%, 0.84 [0.55 to 1.29]) or in adverse events (25.5% vs 22.4%; 1.14 [0.83 to 1.56])
Choi et al. [36]	Any vaping compared with no vaping, not associated with postpartum depression (adjusted for smoking): Any vaping a) in past two years: OR [95% CIs]: 1.09, [0.98–1.29], b) during the three months before pregnancy: 1.00 (0.76–1.32), c) during the last three months of pregnancy: 1.03 (0.73–1.46); Exclusive vaping in past two years: 1.27 (0.88–1.84)		
Nanninga et al. 2023 [47]	Exclusive-smokers, non-users, exclusive-vapers and dual-users compared in a combined analysis: No significant difference for composite of adverse outcomes (*p* = 0.27) (effect sizes not reported) No significant difference for hypertensive disorders (*p* = 0.71), gestational diabetes (*p* = 0.15) or postpartum haemorrhage (*p* = 0.18)	Exclusive-smokers, non-users, exclusive-vapers and dual-users compared in a combined analysis: Exclusive-vapers had higher miscarriage rates than the other groups (*p* = 0.01)	
Rollins et al. 2020 [51]	Vapers (combining exclusive-vapers and dual-users) compared with non-users and exclusive-smokers (not adjusted for smoking and dual-users reported smoking more than exclusive-smokers): Vapers significantly more likely to report depression during pregnancy than non-users (OR [95% CIs]: 4.3 [2.1–8.6]) or exclusive-smokers (2.1 [1.0–4.2])		
Wen et al. 2023 [55]	Low gestational weight gain: Compared with non-users, there was no significant difference for exclusive-vapers adjusted OR [95% CIs]: 0.99 [0.78–1.27] or dual-users: 1.18 [0.96–1.44] (not adjusted for smoking) Compared with exclusive-vapers, exclusive-smokers (1.27 [0.99–1.64]) and dual-users (1.18 [0.86–1.62]) had a similar risk and exclusive-smokers and dual-users had a similar risk (0.93 [0.75–1.15])		
Galbo et al. 2022 [56]	Any vaping during last three month of pregnancy compared with no vaping (not adjusted for smoking): Associated with high maternal blood pressure: OR [95% CIs]: 1.92 [1.72–2.16] No significant associations for gestational diabetes: 1.06 [0.92–1.22] or depression: 1.09 [0.97–1.23]		

In summary, there was no evidence of increased risk for exclusive-vaping compared with non-use for low gestational weight gain [55], postpartum depression [36] or for a composite of adverse outcomes (or for individual outcomes of hypertensive disorders, gestational diabetes or postpartum haemorrhage) [47], and increased risk of miscarriage (possibly a spurious finding due to a sample size of only 10, with three using non-nicotine vapes) [47]. There was evidence of a similar risk for exclusive-vaping vs exclusive-smoking for low gestational weight gain [55] and for a composite of adverse outcomes (and for individual outcomes) [47] and greater risk for exclusive-vaping for miscarriage [47]. The findings for the latter two studies need to be treated with caution as they simultaneously reported no significant difference in risk for exclusive-vaping and non-use. This implies that smoking does not pose an elevated risk, when it has been well established that smoking poses an increased risk [1], and therefore raises questions about the validity of their findings for vaping. One study observed that vapers (combining exclusive-vapers and dual-users) had a greater risk of depression symptoms during pregnancy than non-users or exclusive-smokers [51]. Another study observed increased risk for high maternal blood pressure outcome, but not for gestational diabetes or depression, among those reporting any vaping during the last three months of pregnancy than among non-users [56]. Neither of the latter two studies controlled for smoking status and in one study dual-users reported smoking more than exclusive-smokers [51]. The RCT compared vaping and NRT, with no significant differences for a range of outcomes; adverse effects of vaping may be obscured by higher rates of smoking and high levels of vaping in the NRT group [16]. When the RCT compared those abstinent from smoking and regularly vaping or using NRT with those not using these products, irrespective of trial arm, non-users had significantly more overall adverse outcomes and there were no significant differences for caesarean section rates [57].

#### Biomarkers of exposure

Two studies examined maternal exposure to biomarkers of toxicants and carcinogens during pregnancy and neither reported evidence of increased risk of vaping (see Table 5) [37, 39]. One reported that various toxicant and carcinogen urinary biomarkers were substantially lower for exclusive-vapers than for exclusive-smokers or dual-uses [39], although without statistical tests, and the risk for exclusive-vaping vs non-use was not examined. Levels of the heavy metals lead and cadmium were similar across groups, as expected based on their half-lives. The other study reported that hair biomarkers of carcinogens were not significantly different among dual-users, non-users and exclusive-smokers [37] but did not include exclusive-vapers [37]. Both studies had very small sample sizes, lacked information on the level of exposure to vaping or smoking, and included few biomarkers compared with studies of non-pregnant populations [12, 25], making it difficult to draw conclusions.

**Table 5:** Findings for biomarkers outcomes

Study	Nicotine metabolites	Tobacco specific nitrosamines (TSNA)	Polycyclic aromatic hydrocarbon (PAH) metabolites	Volatile organic compound (VOC) metabolites	Heavy metals
Clemens et al. 2019 [37]	*Geometric mean, 95% CI:* Dual-users’ nicotine levels (11.0, 3.8 to 31.3) and cotinine levels (0.213, 0.006 to 7.672) were significantly higher compared with non-users (1.1, 0.6 to 2.0; 0.000, 0.000 to 0.001); *p* = 0.007, *p* = 0.03, respectively No significant difference for dual-users nicotine or cotinine levels compared with exclusive-smokers: 10.6 6.5 to 17.4, 0.065, 0.009 to 0.465; respectively & *p* = 0.58, *p* = 0.40, respectively	*Geometric mean, 95% CI:* No significant difference in nicotine-derived nitrosamine ketone (NNK) between dual-users (0.030, 0.002 to 0.395) and non-users (0.003, 0.001 to 0.011, *p* = 0.22) or exclusive-smokers (0.131, 0.018 to 0.888, *p* = 0.20) NNAL (NNK metabolite 4-(methylnitrosamino)-1-(3-pyridyl)-1-butanone) levels were not significantly different between dual-users (0.030, 0.002 to 0.395) and non-users (0.004, 0.001 to 0.013, *p* = 0.22) or exclusive-smokers (0.005, 0.001 to 0.025, *p* = 0.20)	NR	NR	NR
Coleman et al. 2021 [39]	During pregnancy, nicotine exposure levels (total nicotine equivalents) were lower for exclusive-vapers compared with dual-users and smokers, and similar for dual-users and exclusive-smokers. Pregnant women had higher nicotine levels than non-pregnant. No statistical comparisons conducted *Geometric mean, 95% CI:* *Pregnant* exclusive-vapers: 0.51, 0.08 to 3.49 Dual-users: 24.88, 14.67 to 42.22 exclusive-smokers: 25.08, 13.69 to 45.95 *Non-pregnant* Exclusive-vapers: 0.44, 0.16 to 1.19 Dual-users: 11.62, 7.80 to 19.50 Exclusive-smokers: 12.33, 7.80 to 19.50	Compared with dual-users and exclusive-smokers, NNAL exposure levels were lower for exclusive-vapers during pregnancy. NNAL was higher for exclusive-smokers than dual-users. Compared with non-pregnant women, pregnant women had higher levels of NNAL. No statistical comparisons conducted *Geometric mean, 95% CI:* *Pregnant* Exclusive-vapers: 14.90, 2.04 to 108.67 Dual-users: 136.80, 80.43 to 232.70 Exclusive-smokers: 196.79, 130.32 to 297.10 *Non-pregnant* Exclusive-vapers: 6.12, 3.59 to 10.41 Dual-users: 125.92, 96.61 to 164.17 Exclusive-smokers: 90.91, 71.58 to 115.45	Compared with dual-users and smokers, 1-hydroxypyrene (1-PYR) and 2-hydroxyfluorene (2-FLU) exposure levels in pregnancy were lower for vapers. 1-PYR was similar for dual-users and smokers. 2-FLU was higher for smokers than dual users Compared with non-pregnant women, except for exclusive-vapers 2-FLU, pregnant women had higher levels of 1-PYR & 2-FLU *Geometric mean, 95% CI:* *Pregnant* Exclusive-vapers: 1-PYR 0.25 (0.14, 0.43) 2-FLU 0.22 (0.15, 0.33 Dual-users: 1-PYR 0.37 (0.24, 0.5) 2-FLU 0.77 (0.54, 1.09) Exclusive-smokers: 1-PYR 0.38 (0.32, 0.45) 2-FLU 0.88 (0.71, 1.07) *Non-pregnant* Exclusive-vapers: 1-PYR 0.18, 0.14 to 0.23 2-FLU 0.23, 0.17 to 0.30 Dual-users: 1-PYR 0.34, 0.30 to 0.39 2-FLU 0.83, 0.70 to 0.98 Exclusive-smokers: 1-PYR 0.29, 0.27 to 0.32) 2-FLU 0.68, 0.58 to 0.78	Compared with dual-users and exclusive-smokers, HPMA and CYMA exposure levels were lower for exclusive-vapers during pregnancy. Both HPMA and CYMA were higher for dual-users than Exclusive-smokers Compared with non-pregnant women, except for Exclusive-vapers HPMA, pregnant women had higher levels of HPMA and CYMA *Geometric mean, 95% CI:* *Pregnant* Exclusive-vapers: HPMA 223.67, 157.69 to 317.25 CYMA 8.48, 3.36 to 21.40 Dual-users: HPMA 1331.07, 974.09 to 1818.86 CYMA 96.74, 66.67 to 140.38 Exclusive-smokers: HPMA 1020.94, 783.61 to 1330.15 CYMA 73.98, 51.20 to 106.88 *Non-pregnant* Exclusive-vapers: HPMA 296.48, 207.92 to 422.67 CYMA 4.36, 2.54 to 7.50 Dual-users: HPMA 861.19, 726.78 to 1020.47 CYMA 82.05, 60.97 to 110.43 Exclusive-smokers: HPMA 697.75, 601.87 to 809.10 CYMA 63.07, 47.96 to 82.93	During pregnancy, exposure to lead and cadmium was similar for exclusive-vapers, dual-users and exclusive-smokers Pregnant women had higher levels of cadmium and lead compared with non-pregnant *Geometric mean, 95% CI:* *Pregnant* Exclusive-vapers: Lead 0.42, 0.17 to 1.00 Cadmium 0.17, 0.04, 0.67 Dual-users: Lead 0.50, 0.35 to 0.71 Cadmium 0.15, 0.09 to 0.22 Exclusive-smokers: Lead 0.49, 0.42 to 0.57 Cadmium 0.22, 0.18 to 0.28 *Non-pregnant* Exclusive-vapers: Lead 0.34, 0.29 to 0.39 Cadmium 0.15, 0.11 to 0.19 Dual-users: Lead 0.40, 0.35 to 0.45 Cadmium 0.17, 0.15 to 0.18 Exclusive-smokers: Lead 0.38, 0.35 to 0.41 Cadmium 0.17, 0.15 to 0.18

*NR* not reported

### Fetal and infant outcomes

The most frequently assessed outcomes were key infant outcomes that have been shown to be detrimentally affected by smoking [1]; namely, preterm birth (PTB)/gestational age (GA), small for gestational age (SGA), and low birth weight (LBW)/birth-weight (BW). Studies also assessed neurological measures, various other fetal and infant outcomes and composite outcomes. The effect sizes for fetal and infant outcomes are presented in Table 6.

**Table 6:** Findings for fetal and infant outcomes

Author/date	Preterm birth (PTB), gestational age (GA)	Birth weight (BW)/ Low birth weight (LBW)	Small for gestational age (SGA)	Neurobehavioural, neuropathological	Other outcomes	
Ammar et al. 2023 [32]	Compared with non-users, neither exclusive vapers nor dual-users (in the last three months of pregnancy) had a significantly increased risk of PTB (aRR, 95% CI: 1.29, 1.00 to 1.65 *(reported in article as significant but this is incorrect as lower CI is 1.0*); aRR, 1.19, 0.97 to 1.48, respectively) Compared with those who continued to vape, quitting vaping during last three months of pregnancy did not significantly reduce risk of pre-term birth (aRR, 95% CI: 0.89, 95% CI: 0.71 to 1.11) Among women not smoking during the three months before pregnancy or in the last three months of pregnancy, quitting vaping was associated with a significantly reduced risk of PTB (aRR 0.68, 95% CI 0.48 to 0.98)	Compared with non-users, both exclusive vapers and dual-users had significantly increased risk of LBW (aRR, 95% CI: 1.38, 1.09 to 1.75; 2.01, 1.63 to 2.48, respectively) When also adjusting for gestational age, compared with non-use, dual-use had significantly increased risk of LBW but exclusive-vaping was no longer significant (aRR, 95% CI: 1.71, 1.42 to 2.06; 1.10, 0.90 to 1.35, respectively). Similarly, when restricting the analysis to full-term births, the association for dual-use remained significant (aRR not reported) but the association for exclusive vaping was not significant (aRR 1.21, 95% CI 0.78 to 1.86) Compared with those who continued to vape, those quitting vaping during last three months had a significantly reduced risk of LBW (aRR, 95% CI: 0.76, 0.62 to 0.94) Among those not smoking during the three months before pregnancy or in the last 3 months of pregnancy, quitting vaping was associated with significantly reduced risk of low birth weight (aRR: 0.60, 95% CI 0.42 to 0.84)	Compared with non-users, dual-users had a significantly increased risk of SGA but exclusive vapers did not (aRR, 95% CI: 2.27 1.90 to 2.72; 1.04, 0.76 to 1.44, respectively) Compared with those who continued to vape, those quitting vaping during last three months had a significantly reduced risk of SGA (aRR, 95% CI: 0.77, 0.62 to 0.94) Among those not smoking during the three months before pregnancy or in the last 3 months of pregnancy, quitting vaping was not associated with significantly reduced risk of SGA (aRR 0.81, 95% CI 0.54 to 1.23)	NR	NR	
Ashford et al. 2021 [33]	No significant differences in GA for those switching between smoking, vaping or dual use during pregnancy (GA mean difference for switching vs not switching: -0.205, *p* = 0.583)	Switching from exclusive-smoking, exclusive-vaping or dual-use to no product use resulted in infants weighing 304g more (*p* = 0.0439) No significant differences in birth weight for those switching between smoking, vaping or dual use during pregnancy (weight mean difference for switching vs not switching: 182.8, *p* = 0.446)	NR	NR	No significant differences in neonatal intensive care unit admissions for those switching between smoking, vaping or dual use during pregnancy (OR, 95% CI: 1.08, 0.47 to 2.50) No significant differences in respiratory distress for those switching between smoking, vaping or dual use during pregnancy (OR, 95% CI: 0.75, 0.26 to 2.21)	
Cardenas et al. 2019 [34]	NR	Compared with non-users, the differences in gender-specific and gestational age-specific birth weight was not significant for vapers or dual-users (Mean z-score BW difference (SE): -0.540 (0.417); -0.303 (0.274), respectively), but it was different for smokers (-0.490 (0.190))	*Relative risk, 95% CI:* Compared with non-users, Relative Risk of SGA was not significantly different for vapers (5.1, 1.2 to 22.2), dual-users (2.5, 0.7 to 8.8) or smokers (2.6, 0.9 to 7.2)	NR	NR	
Cardenas et al. 2020 [35]	NR	NR	*Adjusted Risk ratio (aRR, 95% CI) of SGA:b* Compared with non-users, there were no significant differences in aRR for vapers (RR not calculable due to low sample size), dual-users (1.8, 1.0 to 3.4) or smokers (1.6, 1.0 to 2.6) *Effects of changes in product use between 3 months before pregnancy and last 3 months of pregnancy:* Compared with non-users, there was no significant difference in RR for dual-users who continued smoking at similar level (RR 2.2, 0.6 to 7.4) or who smoked less (RR 1.0, 0.4 to 2.7). There was a significantly increased risk for dual-users who stopped smoking and continued vaping (RR 3.2, 1.5 to 6.6)	NR	NR	
Clemens et al. 2019 [37]	NR	NR	Compared with those not exposed to vaping or smoking, babies of dual-users had a non-significant relative risk, for SGA of 8.3, 95% CI 1.0 to 69.1	NR	NR	
Cohn et al. 2023 [38]	NR	NR	NR	NR	No significant difference in adverse birth outcomes (any incidence of preterm birth, low infant birth weight, baby with birth defects, placenta previa, placenta abruption, or pre-eclampsia) for a group combining past 30-day exclusive vaping and dual-use compared with non-use (Adjusted OR 1.88, 95% CI 0.67 to 5.13)	
Froggatt et al. 2020 [40]	Similar GA for exclusive-vapers (39.84 weeks) and non-users (39.18 weeks)	Birthweight was similar for exclusive-vaping (mean (SD) = 3477.11 (257.91)g) vs non-use (3451.9 (596.69)g)	NR	Neonatal Behavioural Assessment Scale: Compared with non-exposed infants, *abnormal reflexes* significantly higher for exclusive-vaping (d = 1.625, *p* = 0.002) and similar for exclusive-vaping and exclusive-smoking (d = 0.287, *p* = 0.236) Compared with non-exposed infants, *motor maturity* significantly lower for exclusive-vaping (d = 0.732, *p* = 0.036,) and similar for exclusive-vaping and exclusive-smoking (d = 0.103, *p* = 0.745) No significant differences in *self-regulation scores* for non-use vs vaping (d = 0.713, *p* = 0.057) or between smoking and vaping (d = 0.358, *p* = 0.454). Self-regulation scores were significantly lower for smoking vs non-use (d = 0.649, *p* = 0.010)	Mean (SD) cm: Infant head circumference similar for infants of exclusive-vapers (34.4 (0.89) vs exclusive-smokers (33.6 (1.5) or non-users (34.8 (1.5)	
Froggatt et al. 2021 [41]	NR	NR	NR	Ultrasound scans of fetal movements 79 sets of paired 32- and 36-week data When adjusting for time of day, no significant differences in relative frequency of mouth movements between exposure groups (exclusive-vaping, exclusive-smoking, non-use) at 32 or 36 weeks gestation (d = 0.31, *p* = 0.31) Mouth movements significantly declined from 32 to 36 weeks gestation for non-exposed foetuses (*p* = 0.02), but not for the exclusive-vaping (*p* = 0.06) or exclusive-smoking groups (*p* value NR)	NR	
Galbo et al. 2022 [56]	NR	NR	NR	NR	Significantly higher odds of at least one adverse birth outcome among those reporting any vaping during the last three months of pregnancy versus women who did not (aOR 1.62, 95% CI 1.16 to 2.26)	
Hajek et al. 2022 [15] Pesola et al., 2024 [57]	No significant differences between vaping and NRT arms of trial for GA (RR, 95% CI: 0.23, -0.14 to 0.59) or pre-term birth (RR, 95% CI: 0.73, 0.51 to 1.05) Abstainers not using nicotine products (vaping or NRT) had more PTBs than abstainers using these products (aRR, 95% CI: 0.29 (0.12 to 0.70). GA was similar in these groups (mean (SD): 37.6 (4.0), 38.6 (2.7), respectively, RR 95% CI 0.93 (-0.34 to 2.21) Rates of PTB were similar in exclusive smokers (who did not vape or use NRT) and dual-users (11.2%, 9.6%, respectively; RR 95% CI 0.89 (0.60 to 1.34). GA was also similar in these groups (mean (SD): 38.2 (3.2), 38.4 (2.9), respectively, RR 95% CI -0.26 (-0.68 to 0.16)	Compared with babies in vaping arm, babies in the NRT arm had significantly higher relative risk of low birthweight of 0.65 (CI 95% 0.47 to 0.90) BW in smoking abstainers using nicotine products (vaping or NRT) was significantly higher than in smokers (3.3 kg (SD) = 0.7) vs 3.1 kg (SD = 0.6); difference = 0.15 kg, 95% CI: 0.05 to 0.25) and not significantly different from abstainers not using nicotine products (3.1 kg (SD = 0.8) (RR 0.20 (-0.05 to 0.45)) Birthweights in dual-users and exclusive smokers (who did not vape or use NRT) were the same (3.1 kg) Rates of low birth weight were not significantly different for abstainers who were regularly using nicotine products vs those not using these products (9.9%, 16.0%, respectively; RR 95% CI 0.64 (0.24 to 1.72). Nor were they significantly different for dual-users and exclusive smokers (13.2%, 11.3%, respectively; RR 95% CI 1.24 (0.85 to 1.81)	NR	No significant differences between vaping and NRT arms of trial for GA (RR, 95% CI: 0.23, -0.14 to 0.59) or pre-term birth (RR, 95% CI: 0.73, 0.51 to 1.05) Smoking abstainers not using nicotine products (vaping or NRT) had more PTBs than abstainers using these products (aRR, 95% CI: 0.29 (0.12 to 0.70). GA was similar in these groups (mean (SD): 37.6 (4.0), 38.6 (2.7), respectively, RR 95% CI 0.93 (-0.34 to 2.21) Rates of PTB were similar in exclusive-smokers (who did not vape or use NRT) and dual-users (11.2%, 9.6%, respectively; RR 95% CI 0.89 (0.60 to 1.34). GA was also similar in these groups (mean (SD): 38.2 (3.2), 38.4 (2.9), respectively, RR 95% CI -0.26 (-0.68 to 0.16)		*RR, 95% CIs:* No significant differences between vaping and NRT arms of trial for neonatal intensive care unit admissions (1.11, 0.76 to 1.63) or congenital abnormalities (1.68, 0.89 to 3.14) There was insufficient incidence of miscarriage, stillbirth, neonatal death, postnatal death, terminations or maternal death to compute RRs There was insufficient incidence of congenital abnormalities to compute RRs among abstainers. Rates of these abnormalities were not significantly different in dual-users vs exclusive smokers (4.5%, 3.1%, respectively, RR 95% CI 1.38 (0.66 to 2.87)
Hawkins et al. 2021 [42]	Coefficient (95% CI) (adjusted) There was no significant difference in the risk of PTB, compared with non-users, for exclusive-vapers (1.39, 0.84 to 2.30) or dual-users (1.03, 0.73 to 1.46) There was no significant difference in gestational age, compared with non-users, for exclusive-vapers (-0.21, -0.45 to 0.03) or dual-users (-0.15, -0.43 to 0.12)	Coefficient (95% CI) (adjusted) There was no significant difference in birth weight between infants of non-users, and exclusive-vapers (-57.8, -134.3 to 18.6). Infants of dual-users weighed significantly less than those of non-users (-193.9, -274.9 to -112.8, *p* < 0.01)	Coefficient (95% CI) (adjusted) There was no significant difference in the risk of SGA between infants of non-users and exclusive-vapers (0.78, 0.48 to 1.27). Infants of dual-users had a higher risk of SGA than non-users (1.93, 1.31 to 2.83, *p* < 0.01)	NR		NR
Kim et al. 2020 [43]	*Adjusted Odds ratios (OR), 95 CIs:* Compared with non-users, infants of exclusive-vapers were significantly more likely to have PTB (1.86, 1.11 to 3.12) Infants of vapers and smokers had similar odds of preterm birth (1.06, 0.46 to 2.48)	*Adjusted Odds ratios (OR), 95 CIs:* Compared with non-users, infants of exclusive-vapers were significantly more likely to have LBW (1.53, 1.06 to 2.22) Infants of vapers and smokers had similar odds of LBW (0.71, 0.37 to 1.37)	*Adjusted Odds ratios (OR), 95 CIs*: Compared with non-users, infants of exclusive-vapers were significantly more likely to be SGA (1.76, 1.04 to 2.96) Infants of vapers and smokers had similar odds of SGA (0.67, 0.30 to 1.47)	NR		NR
Lavezzi et al. 2022 [44]	NR	NR	NR	Adverse neuropathological brainstem outcomes (pulmonary hypoplasia, pFn hypoplasia) were present in most of those smoking, exclusively vaping and dual-using among cases of sudden intrauterine unexplained deaths. Few non-users had these adverse outcomes. Detailed results NR		NR
Lin et al. 2023 [45]	NR	NR	NR	NR		Adjusted ORs 95% CI: Neither quitting vaping before pregnancy nor vaping (combining exclusive-vapers and dual-users) during pregnancy were significantly associated with high-risk birth (1.14, 0.54 to 2.40; 1.19, 0.38 to 3.73, respectively) or foetal death (0.39, 0.07 to 2.26; 0.33, 0.04 to 2.83 respectively), compared with not vaping *Flavours:* Compared with ‘other flavours’, use of menthol/mint was significantly associated with fetal death (3.27, 1.17 to 9.19), but was not associated with high-risk pregnancy (1.09, 0.22 to 5.49). Neither alcohol nor candy favours were significantly associated with these outcomes vs ‘other flavours’
McDonnell et al. 2019 [46]	Mean gestation at delivery was similar in exclusive vapers, dual-users, smokers and non-users (all 39 weeks)	Babies of exclusive-vapers and non-users had similar BW and incidence of LBW (*p* = 0.97, *p* = 0.60, respectively) Compared with smokers and dual-users, babies of exclusive-vapers had significantly greater BW and lower incidence of LBW (all *p* < 0.001) Babies of exclusive-mokers and dual-users had similar BW and risk of LBW (NR)	Babies of exclusive-vapers and non-users had similar mean birth centile (both 47th centile, *p* = 0.97) Compared with smokers and dual-users, babies of exclusive-vapers had a significantly higher mean birth centile (both *p* < 0.001) Babies of exclusive-smokers and dual-users had a similar mean birth centile (27th & 30th centile, respectively)	NR		Neonatal intensive care unit admissions rates similar for vapers, dual-users, smokers and non-users (range 5% to 8% admitted) APGAR scores were the same for all study groups (all 9, 10)
Nanninga et al. 2023 [47]	There were no significant differences in PTB across the study groups (vapers, smokers, dual-users, non-users) (*p* = 0.75)	NR	Across the four groups, there was an overall significant difference in SGA (0.02), with a tendency for higher rates of SGA in vapers compared with smokers, dual-users and non-users	NR		When comparing the four groups, there was no difference in hospital admissions in the first year of life (*p* = 0.43) or for a composite infant outcome (*p* = 0.11)
Opondo et al. 2021 [48]	Adjusted for smoking pattern, compared with not vaping, vaping (combining vapers and dual-users) was not associated with PTB (risk ratio, 95% CI = 1.29, 0.54 to 2.04, *p* = 0.210) or GA (difference 95% CI = -0.18, -0.58 to 0.21)	Adjusted for smoking pattern, compared with not vaping, vaping (combining exclusive-vapers and dual-users) was not associated with BW (difference 95% CI = -31.7, -125.8 to 62.5)	NR	NR		NR
Regan, Bombard et al. 2021 [49]	Adjusted prevalence ratio, 95% CIs: Compared with non-use, exclusive-vaping in pregnancy had a significantly higher prevalence of PTB (1.69, 1.20 to 2.39) PTB prevalence was similar for exclusive-smokers and dual-users (0.82, 0.59 to 1.14) *Frequency of vaping:* Compared with non-vapers (i.e., group combining smokers and non- users), exclusive-vapers with daily use had a significantly higher prevalence of preterm birth (1.94; 95% CI 1.28 to 2.93). The results was not significant for non-daily use (1.26, 0.71 to 2.22) Prevalence of pre-term birth was similar for ‘non-vapers’ and dual-users both for daily and less than daily vapers	Adjusted prevalence ratio, 95% CIs: Compared with non-use, exclusive-vaping in pregnancy had a significantly higher prevalence of LBW (1.88, 1.38 to 2.57) LBW prevalence was similar for exclusive-smokers and dual-users (1.05 (0.80 to 1.38) *Frequency of vaping:* Compared with non-vapers (i.e., combining smokers and non- users), exclusive-vapers with both daily and non-daily vaping had a significantly higher prevalence of LBW (2.00, 1.34 to 3.00; 1.76, 1.04 to 2.65, respectively) Prevalence of LBW was similar for ‘non-vapers’ and dual-users both for daily and less than daily vapers	Adjusted prevalence ratio, 95% CIs: Compared with non-use, exclusive-vaping in pregnancy was not associated with higher prevalence of SGA (1.10, 0.65 to 1.86) SGA prevalence was similar for exclusive-smokers and dual-users (1.15, 0.89 to 1.48) *Frequency* of vaping*:* Compared with non-vapers (i.e., combining smokers and non- users), prevalence of SGA for exclusive-vapers was similar, irrespective of whether vaping was daily or less than daily (NR) Prevalence SGA was similar for ‘non-vapers’ and dual-users both for daily and less than daily vapers	NR		NR
Regan & Pereira 2021 [50]	*Adjusted prevalence ratios (aPRs,* 95% CI*):* Both exclusive-vapers and dual-users had similar prevalence of PTB to non-users (former smokers) (1.21, 0.78 to 1.87; 1.26, 0.91 to 1.73, respectively) Compared with babies of exclusive-smokers, rates of PTB were similar for both exclusive-vapers and dual-users (0.85, 0.55 to 1.31; 0.88, 0.64 to 1.22, respectively)	*Adjusted prevalence ratios (aPRs,* 95% CI*):* Compared with non-users, both exclusive-vapers and dual-users had higher prevalence of LBW (1.52, 1.01 to 2.29; 2.11, 1.60 to 2.77, respectively) Compared with babies of exclusive-smokers, LBW rates were similar for both exclusive-vapers and dual-users (0.81, 0.54 to 1.21; 1.12, 0.85 to 1.46; respectively)	*Adjusted prevalence ratios (aPRs,* 95% CI*):* Compared with non-users, dual-users had a significantly higher prevalence of SGA (2.60, 2.00 to 3.38) and exclusive-vapers had a similar prevalence of SGA (1.22, 0.63 to 2.34) Compared with babies of exclusive-smokers, SGA rates were similar for both exclusive-vapers and dual-users (0.56, 0.29 to 1.08; 1.20, 0.94 to 1.55)	NR		NR
Shittu et al. 2022 [52]	NR	NR	*Adjusted relative risk (RR), 95% CI:* *Impact of continued use:* Compared with non-users, those exclusive-vaping or dual-using in pregnancy had a significantly increased RR of SGA (1.52, 1.45 to 1.60; 2.53, 2.47 to 2.58, respectively) *Impact of quitting*: Compared with exclusive-vapers pre-pregnancy, exclusive-vapers who quit vaping by late pregnancy had lower SGA risk (0.55, 0.52–0.59) Exclusive-vapers who quit vaping during pregnancy had comparable risk to non-users (RR 0.84, 0.82 to 0.87) Compared with continuous dual-use, quitters of vaping only and of smoking only had a moderate decrease in risk of SGA (0.71, 0.69 to 0.73; 0.60; 0.57 to 0.64, respectively); dual-users who quit both vaping and smoking had the largest decrease in risk (RR 0.48, 0.46 to 0.49) *Impact of switching*: Switching from exclusive-smoking to exclusive-vaping by late pregnancy was associated with a similar risk to non-users (0.83, 0.73 to 0.93) Compared with non-users, pre-pregnancy smokers who continued smoking and started vaping had an elevated risk of SGA (2.09, 2.00 to 2.19) Compared with continuous smokers, continuous smokers who started dual-use had a slightly lower risk of SGA (0.94, 0.90 to 0.98)	NR		NR
Wang et al. 2020 [53]	*Adjusted Odds ratio (aOR), 95% CIs:* Compared with non-users, exclusive-vapers and dual-users had similar risk of PTB (aOR 1.2, 0.5 to 2.7; 1.3, 0.8 to 2.3; respectively)	NR	*Adjusted Odds ratio (aOR), 95% CIs* Compared with non-users, SGA rates for exclusive-vapers were not significantly different (aOR 2.4, 1.0 to 5.7) but were significantly higher for dual-users (2.3, 1.3 to 4.1)	NR		NR
Wang et al. 2022 [54]	*All comparisons in last three months of pregnancy (aOR 95% CIs):* Compared with non-users, the odds of preterm birth was significantly higher for exclusive light smokers (1.27, 1.08 to 1.50) and exclusive heavy smokers (1.45, 1.19 to 1.76), but was not significantly different for exclusive occasional vapers (0.76, 0.35 to 1.65), exclusive frequent vapers (1.21, 0.65 to 2.26), dual-users: light smoker & occasional vaper (0.61, 0.34 to 1.10), dual-users: light smoker & frequent vaper (1.18, 0.58 to 2.40), dual users: heavy smoker & occasional vaper (1.52, 0.80 to 2.89), or dual users: heavy smoker & frequent vaper (1.44, 0.78 to 2.65)	NR	*All comparisons in last three months of pregnancy (aOR 95% CIs):* Compared with non-users, the odds of SGA was significantly higher for exclusive light smokers (2.10, 1.79 to 2.46), exclusive heavy smokers (2.78, 2.34 to 3.29), dual users: light smoker & occasional vaper (1.95, 1.13 to 3.39), dual users: light smoker & frequent vaper (3.37, 1.73 to 6.56), dual users: heavy smoker & occasional vaper (3.40, 2.02 to 5.72), and dual users: heavy smoker and frequent vaper (3.08, 1.33 to 7.10) but was not significantly different for exclusive occasional vapers (1.36, 0.47 to 3.95) or exclusive frequent vapers (1.14, 0.52 to 2.54)	NR		NR

*NR* not reported, *NS* not significant, *SD* standard deviation, *NRT* nicotine replacement therapy, *aPR* adjusted prevalence ratio, *OR* odds ratio, *aOR* adjusted odds ratio, *aRR* adjusted risk ratio

#### Preterm Birth (PTB) and gestational age at birth (GA)

Ten studies reported PTB [16, 32, 42, 43, 47–50, 53, 54] and five reported GA [16, 33, 40, 46, 48]. Nine studies compared exclusive-vapers and non-users and one compared non-users with a group combining exclusive-vapers and dual-users controlling for smoking status [48]. Six of these studies observed no significant group differences in PTB rates [32, 42, 48, 50, 53, 54] and four reported no significant group differences for GA [40, 42, 46, 48]. In one study this was the case irrespective of the frequency of vaping [54]. Conversely, two studies observed significantly greater PTB rates for exclusive-vapers than for non-users [43, 49]. Additionally, one of these studies reported a significantly higher PTB prevalence for ‘daily’ exclusive-vapers than for a group combining exclusive-smokers and non-users, but not for non-daily vapers [49]. Another study found that compared with those who continued to vape, those who quit vaping during pregnancy did not have a significantly lower risk of preterm birth [32]; however, among those who did not smoke during the three months before pregnancy or in the last three months of pregnancy, those who quit vaping had a significantly lower risk of PTB.

Three studies compared exclusive-vaping and exclusive-smoking; one observed a lower risk of GA for exclusive-vaping [46] and two observed a similar risk of PTB for exclusive-vaping and exclusive-smoking [43, 50]. The finding for one of the latter studies should be treated with caution as they also reported no absolute risk for PTB [50]. One study had inconclusive results, observing no significant differences in GA for those switching between exclusive-smoking, exclusive-vaping and dual-use during pregnancy [33]. The RCT found no significant differences in PTB or GA between the vaping and NRT arms [16]. The RCT also found that, irrespective of trial arm, those who reported smoking cessation and the use of NRT or vaping in pregnancy had significantly fewer PTBs than did those reporting cessation and no use of these products [57]; GA was similar in these two groups.

Additionally, six studies compared dual-users with other groups. Five studies observed no significant differences in PTB rates between dual-users and non-users [32, 42, 50, 53, 54] and one observed similar GA for these groups [46]. Three studies compared dual-use and exclusive-smoking and reported similar GA [46] and PTB prevalence [49, 50]. One of the latter studies also found that the prevalence of preterm births was similar for dual-users and ‘non-vapers’ (combination of exclusive-smokers and non-users), irrespective of the frequency of vaping [49]. One study compared exclusive-vaping and dual-use and reported similar GA [46]. Finally, one study compared exclusive-vapers, exclusive-smokers, dual-users and non-users, as a whole, and found no significant difference in PTB rates [47]. The RCT reported that, irrespective of trial arm, exclusive-smokers had rates of PTB and GA similar to those who dual-used smoking in combination with vaping or NRT [57].

To summarise the findings for PTB/GA, eight studies reported that exclusive-vaping did not have a significantly different risk than non-use [32, 40, 42, 46, 48, 50, 53, 54] and two studies reported a significantly higher risk for exclusive-vaping [43, 49]. The finding for the latter two PRAMS studies is inconsistent with five of the above PRAMS studies which found no difference between the groups [32, 42, 50, 53, 54]. This divergence in findings is most likely due to different analyses and inclusion criteria (see Table 1). One study reported that exclusive-vaping had a significantly lower risk than exclusive-smoking [46] and two observed no significant difference between the two groups [43, 49], although one of the latter studies simultaneously found no difference between exclusive-vaping and non-use, which leads us to question the validity of the findings.

#### Small for gestational age/mean birth centile

Small for gestational age (SGA) was reported in 12 studies [32,34–36,42,43,47,49,50,52–54) and mean birth centile (MBC) in one study (46). Eight of ten studies comparing SGA or MBC prevalence between exclusive-vapers and non-users reported no significant differences [32, 34, 35, 42, 46, 50, 53, 54]. In one of these studies, this was the finding irrespective of the frequency of vaping [54]. Two studies observed a significantly higher SGA risk for exclusive-vapers [43, 52]. Consistent with this, one study observed that exclusive-vapers who became non-users by late pregnancy had significantly lower SGA risk than those who continued vaping, and comparable risk to non-users [52]. Similarly, one study reported that, compared with non-users, dual-users who switched to exclusive-vaping had an elevated risk of SGA [35]. Additionally, one study reported that, compared with a group combining exclusive-smokers and non-users, prevalence of SGA for exclusive-vapers was similar, irrespective of whether vaping was daily or less than daily [49].

Three studies compared exclusive-vaping and exclusive-smoking. The first study observed (healthily) significantly higher MBC for exclusive-vapers [46]. The other two studies reported similar SGA rates for the two groups [43, 50]. A further study found that switching from exclusive-smoking to exclusive-vaping significantly reduced the risk of SGA so that it was similar to non-use [52].

Of nine studies comparing dual-use and non-use [32, 34–36, 42, 50, 52–54], six reported a significantly higher prevalence of SGA for dual-users [32, 42, 50, 52–54]. Among four studies comparing dual-use and exclusive-smoking, two observed a similar risk of SGA [49, 50], one observed comparable MBCs [46], and one found that switching from exclusive-smoking to dual-use had little effect on SGA risk [52]. One study compared exclusive-vaping and dual-use and reported a lower MBW for dual-users [46]. A further study reported a significant difference in rates of SGA when comparing exclusive-vapers, exclusive-smokers, dual-users and non-users, as a whole, although pairwise comparisons were not reported [47]. One study showed that quitting vaping during pregnancy vs continuing was not significantly associated with a reduced risk of SGA when controlling for smoking during the three months before pregnancy or in the last 3 months of pregnancy [32].

In summary, for SGA/MBC, eight studies reported that exclusive-vaping did not have a significantly different risk than non-use [32, 34, 35, 42, 46, 50, 53, 54] and two observed that exclusive-vaping had a higher risk [43, 52]. Similar to the findings for PTB/GA, the finding for the latter two ‘PRAMS’ studies is inconsistent with six of the above PRAMS studies which found no group differences [32, 35, 42, 50, 53, 54]. Two studies reported a significantly lower risk for exclusive-vaping than for exclusive-smoking [46, 52] and two reported no significant difference for these two groups [43, 50]. The findings for one of these studies are problematic as they simultaneously observed no difference between exclusive-vaping and non-use.

#### Low birth weight (LBW)/birth-weight (BW)

Eleven studies reported LBW or BW [16, 32–34, 40, 42, 43, 46, 48–50]. Among eight studies comparing exclusive-vaping and non-use, four reported similar BW [34, 40, 42, 46] and four observed a higher likelihood of LBW for infants of exclusive-vapers [32, 43, 49, 50]. In one of these studies the association between exclusive-vaping and LBW was not significant when adjusting for GA or when restricting the analysis to full-term births, indicating that effects on LBW could be mediated by PTB (although the risk of PTB was not significantly associated with exclusive vaping – see above) [32]. One of the studies reported a significantly higher prevalence of LBW for exclusive-vapers than for a group combining exclusive-smokers and non-users, irrespective of whether vaping was daily or less than daily [48].

Among three studies comparing exclusive-vaping and exclusive-smoking, one found lower BW for exclusive smokers [46] and two reported similar LBW risk for the two groups [43, 50]. One study showed that quitting vaping during pregnancy vs continuing vaping was significantly associated with a reduced risk of LBW [32]. However, when focusing the analysis on those who did not smoke during the three months before pregnancy, or in the last three months of pregnancy, this association was not significant.

Of three studies comparing dual-users and non-users, one detected no significant group differences [34], one revealed significantly lower weights for infants of dual-users [42], and the third observed higher risk of LBW for dual-users [32]. Three found similar risk of LBW for dual-users and exclusive-smokers [46, 49, 50]. One study showed that babies of dual-users had significantly lower BW than exclusive-vapers [46]. One study reported that vaping (combining exclusive-vapers and dual-users) was not associated with BW, compared with non-vapers, when adjusting for smoking patterns [48]. A further study found no significant differences in BW for those switching between exclusive-smoking, exclusive-vaping and dual-use during pregnancy but found that those who switched from using any product to no product had infants who weighed significantly more [33]. The RCT found that, compared with the NRT arm, babies in the vaping arm had a significantly lower risk of LBW, most likely due to lower smoking [16]. Additionally, irrespective of trial arm, those who reported smoking cessation and the use of NRT or vaping during pregnancy had similar BWs and LBWs to those reporting cessation and no use of these products and significantly greater BWs than those reporting smoking [57]. BWs and rates of LBWs were similar among those who reported smoking and those reporting dual-use of smoking and vaping or NRT.

In summary, for LBW/BW, four studies reported that exclusive-vaping did not have a significantly different risk than non-use [34, 40, 42, 46], and four reported that exclusive-vaping had a significantly higher risk [32, 43, 49, 50]. For the latter comparisons, as for PTB/GA and SGA/MBC, there are conflicting findings for the five ‘PRAMS’ studies [32, 42, 43, 49, 50]. One study reported significantly lower BW for exclusive-smokers than for exclusive-vapers [46], and two reported no significant difference between exclusive-vaping and exclusive-smoking [43, 50].

#### Infant neurobehavioural and neuropathological outcomes

Three studies reported infant neurological outcomes [40, 41, 44] and compared exclusive-vaping and non-use. The first study observed that abnormal reflex scores were significantly higher and motor maturity scores were significantly lower for infants exposed to vaping [40]. The second reported that, among cases of sudden intrauterine unexplained deaths, adverse neuropathological brainstem outcomes (pulmonary hypoplasia, pFn hypoplasia) were more common for infants of exclusive-vapers [44], although neither samples sizes nor statistical tests were reported. No significant differences in risk were found for exclusive-vaping vs non-use for self-regulation scores [40] or relative frequency of mouth movements [41]. The latter study also observed that mouth movements significantly (and healthily) declined from 32 to 36 weeks gestation for non-exposed fetuses, but not for the fetuses of exclusive-vapers or exclusive-smokers. However, there was a tendency for mouth movements to reduce in the exclusive-vaping group although this group was too small (*n* = 14) to have a good chance of finding a significant difference.

All three studies reported similar levels of adverse neurological outcomes for exclusive-vaping and exclusive-smoking, for the following outcomes: reflex, motor maturity and self-regulation scores [40], relative frequency of mouth movements [41], and neuropathological brainstem outcomes [44]. The finding for self-regulation needs to be treated with caution as they simultaneously reported no heightened risk for self-regulation for exclusive-vaping compared with non-use [40]. One study observed that adverse neuropathological brainstem outcomes were more common for infants who were exposed to dual-use compared with infants of non-users and were similar among infants exposed to exclusive-smoking, dual-use or exclusive-vaping [44].

In summary, among three small studies of neurological outcomes, for at least one outcome, two reported exclusive-vaping having a similar risk to non-use [40, 41] and two reported higher risk for exclusive-vaping [40, 44]. They all reported that exclusive-vaping had a similar risk to exclusive-smoking for all outcomes. These studies had the advantage that they all biochemically verified smoking status and two of the studies assessed the strength of nicotine in the vapes [40, 41].

#### Other fetal and infant outcomes and composite birth outcomes

Eight studies reported other fetal and infant outcomes or composite outcomes [16, 33, 38, 40, 45–47, 56]. Among three studies assessing neonatal intensive care unit (NICU) admissions [16, 33, 46], one observed similar admissions for babies of exclusive-vapers, dual-users, exclusive-smokers, and non-users [46]. The second observed no significant differences in admissions or infant respiratory distress for those switching between exclusive-vaping, dual-use and exclusive-smoking during pregnancy [33]. The RCT found similar congenital abnormalities and NICU admissions for the NRT and vaping arms [16]. When comparing dual-users and smokers, irrespective of trial arm, rates of congenital abnormalities were not significantly different [57].

Nanninga and colleagues examined hospital admissions in the first year of life when comparing babies born to exclusive-vapers, exclusive-smokers, dual-users and non-users, as a whole, and reported no significant difference [47]. Lin et al. found no significant difference in the rates of fetal death between a group combining exclusive-vapers and dual-users vs non-users, controlling for smoking status [45]. This study also observed that, compared with ‘other vaping flavours’, menthol/mint was significantly associated with fetal death but was not associated with a composite of adverse birth outcomes; however, rates of smoking in the different flavour groups were not considered.

Two ‘PATH’ studies reported no significant difference in composite measures of adverse birth outcomes for a combination of exclusive-vapers and dual-users compared with non-users [38, 45], (one study did not control for smoking status [38]) and another found no difference for such a composite when comparing babies of exclusive-vapers, exclusive-smokers, dual-users and non-users, as a whole [47]. Another study observed significantly higher odds of at least one adverse birth outcome among those reporting any vaping during the last three months of pregnancy than among those who did not, although they did not control for smoking status [56].

A further study observed similar infant head circumferences for infants of exclusive-vapers, exclusive-smokers and non-users [40]. One study found that APGAR scores were identical for babies of exclusive-vapers, dual-users, exclusive-smokers and non-users [46].

In summary, there was no evidence of an increased risk of exclusive-vaping compared with non-use for NICU admissions [46] or APGAR scores [46], hospital admissions [47], fetal death [45], a composite measures of adverse birth outcomes [47] or infant head circumferences [40]. There was evidence of similar risk for exclusive-vaping and exclusive-smoking for NICU admissions and APGAR scores [46], hospital admissions [47], a composite measures of adverse birth outcomes [47] and infant head circumferences [40]. Notably, for three of these studies [40, 46, 47] the evidence is questionable as they simultaneously found no increased risk for exclusive-vaping compared with non-use and found similar risk for exclusive-vaping and exclusive-smoking, for the same outcomes.

## Discussion

This review reports on 26 studies examining vaping and health outcomes for pregnant and postpartum women, their fetuses and infants. It focuses on the comparison of exclusive-vaping with non-use of nicotine or tobacco and with exclusive-smoking. The results are complex to interpret as the quality of the studies, except the RCT, was rated as poor, the types of groups compared varied greatly, and for most outcomes there is mixed evidence. Therefore, consistent with the most recent previous review on this topic [27], the findings are inconclusive.

To aid interpretation, we summarise the findings across all studies. For studies comparing exclusive-vaping and non-use, more studies reported no increased risk than reported increased risk. For comparisons of exclusive-vaping and exclusive-smoking, most evidence indicated a similar risk for both groups. Regarding dual-use, for the key fetal and infant outcomes there was the most evidence for dual-use having a comparable risk to exclusive-smoking and presenting an increased risk compared with exclusive-vaping and non-use. Few studies examined dual-use for other outcomes. As none of the studies reported both the frequency and duration of smoking, it is not possible to interpret the implications of the findings for the risks of vaping. Most pregnant women who vape do so to stop smoking or reduce smoking [58]. There is some evidence that vaping may be effective for smoking cessation during pregnancy [16] and effective interventions are needed to help all pregnant people stop smoking, including dual-users of smoking and vaping.

Confidence in the findings is constrained by the many limitations of the studies. Most importantly, none of the studies, except the RCT, adequately assessed exposure to nicotine and tobacco products, especially cigarette smoking. For example, the largest and most rigorous cohort studies used the PRAMS dataset (12 of the included studies), which relies on retrospective, self-reported product use in late pregnancy; therefore, the potential influence of product use during early or mid-pregnancy could not be considered and there was no biochemical verification. Relying on self-reports and lack of assessment of dose may lead to misclassification of exposure, affecting associations with outcomes [59]. Thus, where the risk of exclusive-vaping was reported, as nearly all pregnant women who vape are likely to be current or former smokers [14, 60–62] it is possible that this heightened risk is largely due to high levels of smoking at times beyond the assessment period or concurrent unreported smoking. Additionally, even if smoking were considered, without detailed information on levels of vaping it is not possible to say if more intense vaping poses a risk where no risk of exclusive-vaping was found. These limitations also apply to studies examining switching between products. A further limitation is that, although the 12 PRAMS studies analysed different data sets, with different sample sizes, they had overlapping data, with the same pregnancies included in multiple studies (e.g., they all included data for 2016), which could bias the results. However, for any one outcome, the studies do not always agree (e.g., the findings diverge for the comparison of exclusive-vaping and non-use for the key outcomes of PTB/GA, SGA/MBC and LBW/BW), due to differences in analysis and inclusion criteria, which supports the approach of including them as separate studies.

Other notable limitations included that many studies had small to very small sample sizes for the groups of interest, they did not include the key comparisons of exclusive-vaping with exclusive-smoking and non-use of vaping and smoking and had non-representative samples. The findings of similar risk of exclusive-smoking and exclusive-vaping for six studies, for some outcomes, need to be treated with caution, as they simultaneously found no significantly increased risk for exclusive-vaping compared with non-use [40, 45–47, 50, 54]. This would imply that smoking does not pose an increased risk, which has been established beyond doubt for pregnancy outcomes [1] and raises questions about the validity of their findings on vaping. There were only two studies of biomarkers with small sample sizes, limited study groups and a narrow range of biomarkers.

The sole RCT warrants separate comment. The usefulness of the analysis comparing the randomised groups (vaping vs NRT) is limited for our purposes because most participants continued to smoke and, among those who ceased smoking, only some used the assigned products and some used products assigned to the comparison group. The authors subsequently published a secondary analysis, comparing participants who did or did not use nicotine products (the NRT and vaping groups were combined because there was no indication of differences between them at baseline) regularly during pregnancy, irrespective of randomisation [57]. This secondary analysis reported no evidence of risk for exclusive-vaping, and comparable risk for dual-use (smoking and vaping or NRT) and exclusive-smoking for some outcomes. As the groups were not randomised, unmeasured confounders could influence the findings, but less so than with cohort studies, where groups could differ substantially on factors such as previous smoking levels and when they stopped smoking. In the RCT, participants were reasonably homogenous, in that they smoked daily during early pregnancy, sought help with quitting, were not using nicotine products when recruited, and completed detailed assessments on product use, including biochemical verification, that the cohort studies lacked. The findings are limited in that there were only 25 individuals in the group not using NRT or vaping, and the separate effects of vaping were not examined.

### Implications of the results

In line with recommendations for research on other health effects of vaping [12], large, longitudinal, representative, naturalistic studies examining the risk of vaping are needed, primarily, with women who exclusively vape throughout pregnancy compared with those who have never used nicotine and tobacco products, and with those who exclusively smoke. In addition, studies are needed to examine the risks of vaping among women who smoke and switch to vaping at different stages of pregnancy, when controlling for previous smoking levels, compared with those who smoke or vape throughout pregnancy and those who use no products. Studies also need to identify the risks in women who vape and have never smoked. This research ideally needs to satisfy the following criteria:

- Rigorously assesses self-reports of smoking (of all types of combustible tobacco product) and vaping levels, including the frequency, duration and recency of usage, the type of product used, and nicotine strength.
- Assesses the level of usage of all nicotine and tobacco products (e.g., including NRT)
- Includes biochemical verification of status as an exclusive-vaper or as a non-user of nicotine and tobacco products.
- Conducts assessments at multiple time-points, at pre-pregnancy, during each trimester of pregnancy, in postpartum and beyond.
- Assesses a range of potential confounders of the outcomes, including self-reports of second-hand exposure to smoking and vaping, as well as maternal age, ethnicity, socio-economic status and gestation at the time of the assessments.

Additionally, the risks of vaping need investigating in randomised trials of smoking cessation in pregnancy. Although large cohort studies and RCTs will provide the best data to assess the safety of vaping during pregnancy, these studies are time- and resource-intensive. In the absence of these data from rigorous studies, larger and more rigorously designed biomarker studies are needed to assess the potential harms of vaping during pregnancy. Cross-sectional biomarker studies offer insight into exposure from naturalistic use patterns. Longitudinal research, especially with extended follow-up, is valuable for assessing long-term changes in biomarker exposure, especially among exclusive-vapers with sustained usage, and may be especially important for biomarkers with longer half-lives. Besides the criteria listed above, biomarker studies need to include a broad selection of biomarkers, adequate assessment of exposure to environmental toxins that might affect biomarkers (e.g., occupational exposure to acrolein, air pollution), and consideration of the half-lives of biomarkers.

As noted previously [12], there is a need for standardisation of methodologies among studies exploring the health risks of vaping, including in pregnancy, to aid synthesis and comparisons across studies. Standardisation is especially needed in the assessment of the levels of vaping and smoking, and in the type of maternal, fetal and infant outcomes assessed, including biomarkers of exposure. Greater user engagement and involvement in this research is needed. Pregnant women who currently or previously vape or smoke can help to design research to ensure it is addressing relevant questions, to assist with interpreting findings and to aid dissemination.

### Strengths and limitations

The strengths of this review include the use of broad inclusion criteria, the identification of a large number of recent studies covering a wide range of outcomes, with several researchers involved in screening and data extraction, and the inclusion of a thorough quality assessment. Limitations include the exclusion of non-English language studies and it only being possible to present a narrative review rather than a meta-analysis, as there were insufficient high-quality studies.

## Conclusions

Recommendations around the use of vaping in pregnancy for harm reduction can be informed by studies examining the evidence for the risks of vaping for the fetus, mother, and infant. The evidence for the effects of smoking on these outcomes is well established for non-pregnant smokers versus non-smokers.^1^ A challenge with research establishing health consequences of vaping in pregnancy is that most vapers are former or current smokers and in the studies reporting risks of vaping it is not clear whether the harms were caused by smoking. A related challenge is the uncertainty inherent in self-reporting of vaping and smoking, particularly in this population, and the lack of detailed assessments, such as the amount, frequency and duration of use and composition, including nicotine strength, of products. The evidence is also uncertain from studies reporting no risk of vaping because the levels and content of vaping are not known. Overall, more studies found no evidence of increased risk for exclusive-vaping vs non-use than detected a risk, and more observed comparable risk for exclusive-vaping and exclusive-smoking than observed lower risk for exclusive vaping. These two sets of observations are somewhat contradictory and are likely due to the poor quality of the evidence, which continues to limit confidence in conclusions. Overall, this review confirms the findings of a previous review [27] that the studies are of poor quality, which disallows the use of meta-analysis, and the findings are inconclusive.

### Supplementary Information

Supplementary Material 1.

## Data Availability

The datasets supporting the findings of this article are included within the article and within articles included in the review.
