# Supplementary material for: Vaping during pregnancy: a systematic review of health outcomes
Source: BMC Pregnancy Childbirth. 2024 Jun 20;24:435. doi: 10.1186/s12884-024-06633-6 (PMC11191278; doi:10.1186/s12884-024-06633-6)
Supplement: Supplementary file 1 — Supplementary Material 1. [file 12884_2024_6633_MOESM1_ESM.docx]

**Additional file 1. Search Strategies**

We searched MEDLINE, Embase, PsycInfo, Cumulative Index to Nursing and Allied Health Literature (CINAHL), Maternity and Infant Care, and the Cochrane Central Register of Controlled Trials.

**Medline** (using OVID platform)

| 1 | exp pregnancy/ |
| --- | --- |
| 2 | (pregnan* or prenatal or pre-natal or antenatal or ante-natal or perinatal or peri-natal or postnatal or post-natal or postpartum or post-partum).ti,ab. |
| 3 | (foetus* or fetus* or newborn* or "new-born*" or neonate* or infant* or baby or babies).ti,ab. |
| 4 | exp infant/ |
| 5 | 1 or 2 or 3 or 4 |
| 6 | exp vaping/ |
| 7 | (vaping or vape* or "electronic cigarette*" or e-cig* or "electronic nicotine delivery system*" or "electronic delivery system*" or “ENDS”).ti,ab. |
| 8 | 6 or 7 |
| 9 | 5 and 8 |
| 10 | exp animal/ not human/ |
| 11 | 9 not 10 |
| 13 | limit 11 to english language |

**Embase** (using OVID platform)

001 exp pregnancy/

002 (pregnan* or prenatal or pre-natal or antenatal or ante-natal

or perinatal or postnatal or post-natal or postpartum or

post-partum or foetus* or fetus* or newborn* or "new-born*"

or neonate* or infant* or baby or babies).ti,ab.

003 exp infant/

004 1 or 2 or 3

005 exp vaping/

006 (vaping or vape* or "electronic cigarette*" or e-cigarette*

or e-cig* or "electronic nicotine delivery system*" or

"electronic delivery system*").ti,ab.

007 5 or 6

008 4 and 7

009 8

010 limit 9 to human

**PsycInfo** (using OVID platform)

| 1 | exp pregnancy/ |
| --- | --- |
| 2 | (pregnan* or prenatal or pre-natal or antenatal or ante-natal or perinatal or peri-natal or postnatal or post-natal or postpartum or post-partum).ti,ab. |
| 3 | (foetus* or fetus* or newborn* or "new-born*" or neonate* or infant* or baby or babies).ti,ab |
| 4 | exp infant development/ |
| 5 | exp Electronic Cigarettes/ |
| 6 | (vaping or vape* or "electronic cigarette*" or e-cig* or "electronic nicotine delivery system*" or "electronic delivery system" or “ENDS”).ti,ab. |
| 7 | 1 or 2 or 3 or 4 |
| 8 | 5 or 6 |
| 9 | 7 and 8 |
| 10 | limit 9 to human |
| 11 | limit 9 to animal |
| 12 | 10 not 11 |
| 13 | limit 12 to english language |

**Cumulative Index to Nursing and Allied Health Literature (CINAHL)** (using EBSCO platform)

| S13 | S9 and S12 |
| --- | --- |
| S12 | S11 not S10 |
| S11 | (MH “Human”) |
| S10 | (MH “Animals+”) |
| S9 | S5 and S8 |
| S8 | S6 or S7 |
| S7 | TI (vaping or vape* or “electronic cigarette*” or e-cig* or "electronic nicotine delivery systems" or ENDS) OR AB (vaping or vape* or “electronic cigarette*” or e-cig* or "electronic nicotine delivery systems" or ENDS) |
| S6 | (MH "Vaping") |
| S5 | S1 or S2 or S3 or S4 |
| S4 | (MH "Infant+") OR (MH "Infant, Newborn, Diseases+") |
| S3 | TI (foetus* or fetus* or newborn* or new-born or neonate* or infant* or baby or babies) OR AB (foetus* or fetus* or newborn* or new-born or neonate* or infant* or baby or babies) |
| S2 | TI (pregnan* or prenatal or pre-natal or antenatal or ante-natal or perinatal or peri-natal or postnatal or post-natal or postpartum or post-partum) OR AB (pregnan* or prenatal or pre-natal or antenatal or ante-natal or perinatal or peri-natal or postnatal or post-natal or postpartum or post-partum) |
| S1 | (MH "Pregnancy+") |

**Maternity and Infant Care** (using MIDIRS platform)

| 1 | (pregnan* or prenatal or pre-natal or antenatal or ante-natal or perinatal or peri-natal or postnatal or post-natal or postpartum or post-partum).ti,ab. |
| --- | --- |
| 2 | (foetus* or fetus* or newborn* or "new-born*" or neonate* or infant* or baby or babies).ti,ab |
| 3 | (vaping or vape* or "electronic cigarette*" or e-cig* or "electronic nicotine delivery system*" or "electronic delivery system" or “ENDS”).ti,ab. |
| 4 | 1 or 2 |
| 5 | 3 and 4 |

**Cochrane Central Register of Controlled Trials**

| #1 | MeSh descriptor: [Pregnancy] explode all trees |
| --- | --- |
| #2 | MeSh descriptor: [Infant] explode all trees |
| #3 | MeSh descriptor: [Fetus] explode all trees |
| #4 | #1 or #2 or #3 |
| #5 | MeSh descriptor: [Vaping] explode all trees |
| #6 | #4 and #5 |
